# Deciphering the Role of NLRP-3/Caspase-1/GSDMD Pyroptotic Signal, miR-675-5p, and miR-1247-5p in Mitigation of Neurobehavioral and Neuropathological Alterations in Rotenone-Induced Striatal Neurodegeneration by *Vitex agnus-castus* Leaf Extract and/or Pramipexole in Male Rats

**DOI:** 10.1007/s12035-025-05405-3

**Published:** 2025-12-06

**Authors:** Hebatallah M. Saad, Kariman A. Esmail, Duaa Eliwa, Neveen R. Ashoura, Asmaa A. Aboushouk, Omnya Elhussieny, Hanan A. Edres, Aya H. Rohiem

**Affiliations:** 1 Department of Pathology, Faculty of Veterinary Medicine, Matrouh University, Matrouh, Egypt; 2 Department of Physiology, Faculty of Veterinary Medicine, Alexandria University, Alexandria, Egypt; 3 Department of Pharmacognosy, Faculty of Pharmacy, Tanta University, Tanta, Egypt; 4 Department of Pharmacology, Faculty of Veterinary Medicine, Alexandria University, Alexandria, Egypt; 5 Department of Pathology, Faculty of Veterinary Medicine, Alexandria University, Alexandria, Egypt; 6 Department of Histology and Cytology, Faculty of Veterinary Medicine, Matrouh University, Matrouh, Egypt; 7 Department of Biochemistry, Faculty of Veterinary Medicine, Alexandria University, Alexandria, Egypt

**Keywords:** Rotenone, Vitex agnus-castus, Pramipexole, Pyroptosis, Brain

## Abstract

Rotenone (ROT ) exposure causes behavioral and motor abnormalities, including bradykinesia, catalepsy, and unsteady gait, as in Parkinsonism. *Vitex agnus-castus* (*Vitex A-C*) has been extensively utilized in the management of various female ailments besides its role as an agonist for D2 dopaminergic receptors. Pramipexole (Prami) is a dopamine agonist (DA) receptor , which can reduce complications of dopamine therapy. Therefore, this investigation aimed to assess the possible ameliorating effects of *Vitex A-C* and/or Prami against ROT-evoked striatal neurodegeneration, as well as to shed light on the possible underlying mechanisms . Seventy adult male albino rats were allocated into seven groups (*n* = 10 rats/group): Group I (control group), Group II (Prami control group), Group III (*Vitex A-C* control group), Group IV (ROT group), while Groups V–VII were injected with an intraperitoneal injection of ROT along with a daily oral administration of *Vitex A-C* orPrami, or their combination, respectively, for 60 days. Molecular docking results showed that Vitexin complements with superior performance in α-synuclein (− 5.3 vs − 3.4 kcal/mol), caspase-1 (− 6.9 vs − 4.6 kcal/mol), and NF-κB p65 (− 6.8 vs − 4.5 kcal/mol) targeting. Agnuside’s dopaminergic and anti-inflammatory effects, with Vitexin’s anti-aggregation and anti-inflammatory properties. Our results indicated that *Vitex A-C* and/or Prami markedly ameliorated ROT-induced striatal neurodegeneration, evidenced by their abilities to mitigate ROT-triggered neurobehavioral alterations, dopamine, oxidative stress (MDA), antioxidant (GPX and catalase), and inflammatory markers (NF-kB P65, IL-1β). *Vitex* *A-C* and/or Prami-treated groups decreased pyroptotic signal as evidenced by a remarkable decline in the protein expression of NLRP3, caspase-1, and GSDMD; gene expression of ASC; and tissue levels of IL-1β and IL-18. Additionally, *Vitex A-C* and/or Prami substantially downregulated α-synuclein and upregulated TH protein expressions. On the molecular levels, the combination group rectified ROT-triggered dysregulations in the expressions of HMGB1, AIF-1, and miR-1247-5p without any significant impact on miR-675-5p. The combined therapy showed an improvement in striatal histoarchitecture with mitigation of caspase-1 and glial fibrillary acidic protein immunoreactivity with an upregulation in synaptophysin immunoreactivity. In conclusion, the combined therapy of *Vitex A-C* and Prami holds a promising therapeutic avenue over them alone against ROT-associated striatal neurodegeneration via inhibiting the pyroptotic pathway.

**Graphical Abstract:**

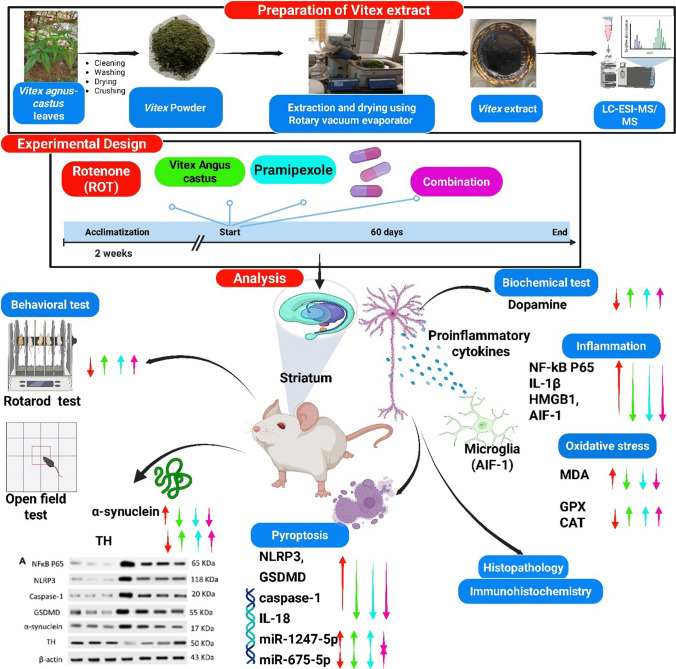

**Supplementary Information:**

The online version contains supplementary material available at 10.1007/s12035-025-05405-3.

## Introduction

Pesticides are environmental poisons that target the central nervous system, and their repeated exposure may lead to neurological disorders such as Alzheimer’s disease and Parkinson’s disease (PD) [1]. A naturally occurring pesticide, rotenone (ROT), is mostly obtained from the stems and roots of the *Lonchocarpus* and *Derris* plant species [2]. In veterinary medicine, ROT powder is used to manage parasitic mites in chickens and other poultry, as well as ticks and lice on horses, dogs, and cats [3]. Experimental research indicated that exposure to ROT led to behavioral and motor impairments, encompassing muscular rigidity (catalepsy), bradykinesia, postural instability, and unsteady gait [4, 5]. Furthermore, ROT is used as an animal model for Parkinsonism because it has been demonstrated in multiple studies to cause α-Synuclein aggregation and phosphorylation, oxidative stress, decreased feeding activities, impaired motor activities, decreased tyrosine hydroxylase (TH) expression, and dopamine secretion in addition to mitochondrial dysfunction [5–7]. ROT is a potent inhibitor of the mitochondrial respiratory chain’s complex I. It works by preventing electrons from moving from the complex’s iron-sulfur centers to ubiquinone, which stops oxidative phosphorylation and limits the amount of ATP produced [8, 9]. Furthermore, this pesticide causes an excessive production of reactive oxygen species (ROS), which causes serious oxidative damage to proteins, lipids, and DNA and triggers dopaminergic neurons to undergo apoptosis [10].

Despite a number of hypotheses about the pathophysiology of PD, neuronal death brought on by neuroinflammation is receiving a lot of attention. Microglial cells and their inflammatory cytokines, such as interleukin (IL)−1β, play a critical role in the development of PD. The activation of the inflammasome and the inflammatory cell death process known as pyroptosis are the main processes that control the release of IL-1β from microglia [11, 12]. Pyroptosis, an inflammatory form of programmed cell death mediated by the NOD-like receptor protein 3 (NLRP3) inflammasome, caspase-1 activation, and Gasdermin D (GSDMD) cleavage, is a key pathological contributor to dopaminergic neuronal injury [13, 14]. Several studies proposed that NLRP3 suppression may improve α-synuclinopathy and stop dopaminergic neuron degeneration [15, 16]. In PD, NLRP3 suppression is a potentially effective disease-modifying strategy. OLT1177 and Selnoflast, two more recent medicines, exhibit translational potential, while MCC950 verified the target but had toxicity problems [17–20].

Emerging evidence also points to microRNAs (miRNAs) as critical regulators of neuroinflammation and neuronal survival. Specifically, miR-675-5p and miR-1247-5p have been implicated in modulating inflammatory responses and cellular resilience in neurodegenerative conditions [21, 22]. Thus, understanding the molecular mechanism of ROT-induced neurotoxicity might reveal novel paths for PD therapeutics.

Nowadays, phytochemicals are excellent alternatives to chemicals and drugs, which are often associated with negative side effects [23]. The chaste tree, *Vitex agnus-castus* L. (*Vitex A-C*), is a medicinal plant that is native to the Mediterranean area and has spread throughout Asia, Europe, and North Africa. It is a member of the *Verbenaceae* family [24]. Historically, *Vitex A-C* fruit extract has been extensively utilized in the management of various female ailments, including menstrual irregularities, premenstrual syndrome, and cyclic mastalgia [25]. Additionally, this plant possesses health-promoting and nutraceutical potential, including antimutagenic, antioxidant, antibacterial, cytotoxic, antiepileptic, immunomodulatory, anti-inflammatory, and antinociceptive activities [26–28]. In rats with lipopolysaccharide (LPS)-induced Alzheimer-like symptoms, *Vitex A-C* extract significantly reduces oxidative stress indices and inflammatory cytokines, as well as improves memory and cognition [29]. Such a beneficial effect seemed probably due to the presence of bioactive ingredients, including flavonoids (Casticin, quercetagetin, and isovitexin), essential oils, diterpenes, and glycosides [28].

Pramipexole (Prami), a non-ergot-derived dopaminergic agonist, is approved by the Food and Drug Administration for the treatment of PD and restless leg syndrome (RLS). By stimulating the striatal pre-synaptic D2 and D3 receptors, Prami reduces the synthesis, release, and renewal of dopamine, which alleviates stiffness, resting tremor, and dyskinesia. Prami’s affinity for D3 is around eight times that of D2 [30]. Aside from dopaminergic activity, Prami has a limited affinity for several adrenergic and serotonergic receptors [31]. Prami may enhance neuroprotection in brain injury through antioxidant mitochondrial pathways [32]**.** By attaching itself to presynaptic dopamine autoreceptors, it negatively influences endogenous dopamine production. This process lessens oxidative stress, which lessens the harm to the nigrostriatal circuits [33], leading to alleviation of dyskinesia, rigidity, and resting tremor [30]. Prami treatment dramatically alleviated the loss of dopaminergic neurons and decreased the levels of IL-1β and astrocyte activation in the substantia nigra with the reduction of NLRP3 inflammasome-associated proteins, such as caspase-1 and ASC, in LPS-injected mice [34].

Therefore, this study seeks to clarify the neuroprotective potential of *Vitex A-C* and Prami (a selective dopamine receptor agonist) against a ROT-induced rat model of neuronal injury. By examining the modulation of the NLRP3/caspase-1/GSDMD pyroptotic axis and the expression profiles of miR-675-5p and miR-1247-5p, this work provides new insights into the molecular mechanisms underlying their effects and advances the therapeutic prospects for PD and related neurodegenerative disorders.

## Materials and Methods

### Preparation and Analysis of *Vitex agnus-castus*

1. Plant Materials
  Samples of fresh and healthy leaves of *Vitex agnus-castus* L*.* were gathered from the plantation of Tanta University, Tanta city, Al-Gharbia gove, Egypt. It was identified by a staff member of the Botany Department, Faculty of Science, Tanta University, Tanta. A voucher specimen (PG-12–5-HD2) was deposited in the Pharmacognosy Department herbarium at Tanta University, Tanta.
2. Preparation of Crude Vitex agnus-castus L. Leaves Extract
  The leaves were cleaned manually, then washed with water to remove dust particles, and shade dried, and then, it was further ground to a fine powder by a grinding machine. Vitex leaves powder (600 g) was extracted using 90% methanol (3 times × 6 L, 72 h each) using the maceration method at ambient temperature (approximately 25 °C) under constant shaking and then filtered by passing through a filter paper. After that, the *Vitex agnus-castus* methanolic filtrate obtained was evaporated and concentrated to dryness by a rotary evaporator under pressure**,** at 40 °C to obtain a dried extract (15 g, 2.5% w/w), transferred to containers, and prepared for further phytochemical and biological assays.
3. LC-HRMS/MS
  The LC-tandem MS (LC-HRMS/MS) analysis of the prepared *Vitex* leaves extract was performed in the Proteomics and Metabolomics Unit, Children’s Cancer Hospital (57,357), according to previously reported methods [35, 36].

### Molecular Docking

1. Ligand Preparation
  The structures of Ligands were downloaded from the PubChem database. The 3D structure was energy-minimized using Avogadro 1.2.0 software [37] with the MMFF94 force field.
2. Protein Preparation
  The UniProt database was used to retrieve the 3D structure proteins (Alpha-synuclein-P37377, Casp1-P43527, dopamin_receptor-P30729, GSDMD-A0A096MJ11, NFKB_p65-Q7TQN4, NLRP3-D4A523, and tyrosine_hydroxylase-P04177). The binding sites for these proteins were predicted using information from the literature and confirmed using the CB-DOCK2 [38]. The proteins were docked using AutoDock Tools 1.5.7 [39], which included polar hydrogen additions, the removal of water molecules, and the assignment of Gasteiger charges.
3. Molecular Docking
  AutoDock Vina [40] was used to conduct molecular docking experiments in order to estimate the compounds’ affinities and mechanisms of binding with each protein. The projected binding locations served as the core of the docking grid boxes. For the docking computations, the default scoring algorithm was used, and the exhaustiveness parameter was set to 8.
4. Visualization and Analysis
  BIOVIA Discovery Studio Visualizer 2021 was used to visualize and evaluate the docked complexes [41]. Intermolecular interactions, such as hydrogen bonds and hydrophobic interactions, as well as binding affinities (Δ*G* values), were examined and documented.

### ADMET

Admet analysis and pharmacokinetics were performed using ADMETLab2.0 [42].

### Ethical Statement

The Institutional Animal Care and Use Committee (IACUC), Alexandria University, Egypt, approved the experimental methods under authorization code ALEXU-IACUC, 013–2025-13–01/335. Every technique followed the ARRIVE guidelines [43].

### Experimental Design

At 8 weeks of age, 70 healthy male albino rats with an average weight of 200 g were kept at the Department of Physiology animal house at the Faculty of Veterinary Medicine after being acquired from Alexandria University’s Medical Research Institute. The rats were kept in plastic boxes with a sawdust bed and a natural dark–light cycle at 21 °C ± 2 °C and 60% ± 2% humidity. The mattress is replaced every day to keep the area dry and hygienic. The rats were disease-free and given unrestricted access to a typical diet and water.

Rats were weighed and divided into seven equal groups (*n* = 10) at random after 2 weeks of housing as following:

- Group I: Control group: received 1 mL of saline orally and intraperitoneally injected with 0.5 mL saline dissolved in 2% dimethyl sulfoxide (DMSO).
- Group II: Control pramipexole group (Prami): orally received 0.5 mg/kg of Prami dissolved in 1 mL saline [44].
- Group III: *Vitex agnus-castus* L. control group (*Vitex A-C*): orally received 165 mg/kg of vitex extract [45, 46].
- Group IV: rotenone group (ROT): IP injected with 3 mg/kg of ROT, which is dissolved in 2% DMSO [6, 47].
- Group V: (ROT + *Vitex A-C* extract group): received both ROT and *Vitex A-C* at previously described doses.
- Group VI: (ROT + Prami group): received both ROT and Prami at previously described doses.
- Group VII: Combination group: received ROT, *Vitex A-C* extract, and Prami at previously described doses. All treatment protocols were followed daily for 60 days.

### Neurobehavioral Tests

1. Rotarod Test
  Each rat was placed on the cylinder of the rotarod apparatus, and the time was measured at a constant speed of 15 rpm for 3 min. Three trials were conducted on the rod, and the mean duration was recorded [48].
2. Open Field Test
  The floor was split into squares (20 cm × 20 cm), and each rat was positioned in the middle of an open field box that measured 1 m × 1 m × 50 cm. For 5 min, locomotor activity was monitored in a calm room with regulated lighting while the rat was positioned in the middle of the field. Between each rat, 70% ethanol was used to wipe the box’s floor. A video camera was used to capture the following parameters: rearing, grooming, center crossing, squares crossed, and latent period. Prior to testing, rats were not given any instructions [49].

### Sample Collection

After 60 days, animals were anesthetized with isoflurane and sacrificed by cervical dislocation to collect the striatal tissue for further biochemical, histopathological, and molecular studies.

#### Biochemical Analyses in the Striatum

1. Dopamine Assessment
  Dopamine was assessed quantitatively in striatal homogenate using a rat dopamine ELISA Kit from CUSABIO Co. (CSB-E08660r). It was expressed as ng/mg protein.
2. Oxidative Stress Biomarker Assessment
  Malondialdehyde (MDA) was measured in the striatal homogenate according to the thiobarbituric acid (TBA) method described by Draper and Hadley [50]. Also, glutathione peroxidase (GPx) activity was assessed at 340 nm as described previously by Flohé and Günzler [51], depending on the production of reduced glutathione (GSH) under the effect of glutathione reductase enzyme on the oxidized glutathione (GSSG) in the presence of NADPH**.** The GPX activity was expressed as mU/mg protein. In addition, catalase (CAT) activity was measured at 240 nm as recorded by Beers and Sizer [52]. The CAT activity measures the amount of CAT that catalyzes the H_2_O_2_ to H_2_O and O_2_ radicals. One unites of CAT activity decomposes one micromole of H_2_O_2_/minute and is expressed as U/mg protein.
3. Some Proinflammatory Cytokines Assessment
  The levels of both interleukin-1β (IL-1β) and interleukin-18 (IL-18) were quantitatively evaluated in striatal homogenate using rat-specific ELISA kit (CUSABIO CO., catalogue nos. CSB-E08055r and CSB-E04610r, respectively). They were expressed by pg/mg protein. All biochemical parameters were evaluated in accordance with the guidelines provided by the manufacturer.
  *Determination of Protein Expressions of NFkB* p65*, NLRP3, GSDMD, Caspase-1, α-Syn, and TH in Rat’s Striatum Using Western Blotting Analyses.*
  The protein expression levels of NFκB p65, NLRP3, GSDMD, caspase-1, α-syn, and TH proteins were evaluated by Western blotting. An ice-cold lysis buffer with a protease inhibitor cocktail (pH 6.8) was used to lyse striatum homogenates. In accordance with the manufacturer’s instructions, protein extraction was carried out using the ReadyPrepTM Total Protein Extraction Kit (Bio-Rad, catalogue no #163–2086). Using the Bradford Protein Assay Kit (Bio Basic, catalogue no. #SK3041), protein concentrations were measured [53].
  Twenty micrograms of each protein sample was combined with an equal volume of 2 × Laemmli sample buffer, which contained 4% sodium dodecyl sulfate (SDS), 10% 2-mercaptoethanol, 20% glycerol, 0.004% bromophenol blue, and 0.125 M Tris HCl (pH 6.8), in order to perform sodium dodecyl sulfate–polyacrylamide gel electrophoresis (SDS-PAGE). Before being loaded onto the polyacrylamide gel, which was made using the TGX Stain-FreeTM FastCastTM Acrylamide Kit (Bio-Rad, catalogue no #161–0181), the mixture was heated for 5 min at 95 °C to denature the proteins.
  The Bio-Rad Trans-Blot Turbo Transfer System (Bio-Rad, catalogue #170–4155) was used to transfer proteins onto a polyvinylidene fluoride (PVDF) membrane. The transfer parameters were adjusted to 25 V for 7 min in a 1 × transfer buffer (25 mM Tris, 190 mM glycine, 20% methanol). For 1 h at room temperature, the membrane was blocked in tris-buffered saline with Tween 20 (TBST) that included 3% bovine serum albumin (BSA). A total of 20 mM Tris (pH 7.5), 150 mM sodium chloride (NaCl), 0.1% Tween 20, and 3% BSA made up the blocking buffer.
  According to the manufacturer’s instructions, TBST was used to prepare the primary antibodies for NFκB p65 (catalogue no #sc-8008, 65 kDa, Santa Cruz), NLRP3 (catalogue no #HY-P80246, 118 kDa, MedChemExpress), GSDMDC1 (catalogue no #NBP2-33,422, 53 kDa, NOVUS), caspase-1 p20 (Cleaved Asp296) (catalogue no #PA5-99,390, Invitrogen), α-syn (catalogue no #0842–1-AP, 14 kDa, Proteintech), and TH (catalogue no #25,859–1-AP, 59 kDa, Proteintech). To identify the housekeeping protein β-actin, an anti-β-actin antibody (Abcam, Catalogue #ab8224) was used. The primary antibodies were incubated on the membranes for a whole night at 4 °C. The membranes were rinsed three to five times with TBST before being treated for 1 h at 24 °C with a secondary antibody conjugated with horseradish peroxidase (HRP) (Goat anti-rabbit IgG-HRP, Novus Biologicals, catalogue no #NBP2-30347H). After incubation, the membranes were rinsed three to five times with TBST, and a ChemiDocTM Imaging System (Bio-Rad, catalogue no #170–8281) was used to see the protein bands. Each band’s densitometric intensity was measured and adjusted for the housekeeping gene’s expression.

###  Real-Time Quantitative (qRT-PCR) Analysis of AIF-1, HMGB1, andASC

 The mRNA expression of allograft inflammatory factor 1 (AIF-1), high mobility group box 1 protein (HMGB1), and apoptosis-associated speck-like protein containing a CARD (ASC) was assessed in the striatum using the RT-PCR method. The QuantiTect Reverse Transcription Kit Catalogue no. 205311 (Qiagen, Germany) was used to reverse-transcribe the total mRNA into complementary DNA (cDNA) after it had been extracted using the miRNeasy Mini Kit according to the manufacturer’s guidelines. As shown in Table 1, the cDNA amplicons were amplified using the ViPrime PLUS Taq qPCR Green Master Mix I (Vivantis Technologies, Malaysia, catalogue number QLMM12) and specific primers. An Applied Biosystems real-time PCR device (Bio-Rad Laboratories, CA, USA) was used to measure the amounts of mRNA transcripts. With β-actin serving as the housekeeping gene, the 2^−ΔΔCt^ technique, as outlined by Livak and Schmittgen [55], was used to compute the relative gene expression or fold changes.

**Table 1:** Primer sets of assayed genes and 18 s rRNA

Gene	Accession No.	primer sequence		
**ASC (PYCARD)**	NM_172322.2	**F**	CTGCTCAGAGTACAGCCAGAAC	
		**R**	CTGTCCTTCAGTCAGCACACTG	
**HMGB1**	NM_012963.4	**F**	CCAAGAAGTGCTCAGAGAGGTG	
		**R**	GTCCTTGAACTTCTTTTTGGTCTC	
**AIF-1**	NM_017196.3	**F**	TCTGCCGTCCAAACTTGAAGCC	
		**R**	CTCTTCAGCTCTAGGTGGGTCT	
** 18 s rRNA**	NR_046237.2	**F**	GTAACCCGTTGAACCCCATT	
		**R**	CAAGCTTATGACCCGCACTT	

###  Relative Quantification of miR-675-5p and miR-1247-5p Expressions Using qPCR

 The TaqMan® kit (Thermo Fisher Scientific, catalogue no. 4427975, ID: 001940, and 002893, respectively) was used to measure the miR-675-5p and miR-1247-5p expressions in the striatum. The CFX96 Touch Deep Well Real-Time PCR Detection System (Bio-Rad Laboratories, CA, USA) was used for amplification, data acquisition, and analysis. The CFX Maestro Software version 1.1 (Bio-Rad Laboratories, CA, USA) was used to calculate the threshold cycle (Ct) values, and the corresponding variations in miR-675-5p and miR-1247-5p in specimens were calculated using the 2^−ΔΔCt^ method and normalized to the reference described [55].

###  Histopathological Assessments

 The brain was carefully excised, then immediately placed in 10% neutral buffered formalin fixative for 48 h, then dehydrated, deparaffinized, and sectioned by rotatory microtomes with 4 µm thickness . Then, brain sections undergo hematoxylin and eosin (H&E) staining procedures [56]. The semiquantitative neuronal loss, gliosis, and vacuolization score was evaluated in five randomly chosen fields from every animal. According to the severity of lesion, the striatal tissue is graded using the following three categories: 0 = no lesion, 1 = mild, 2 = moderate, and 3 = marked [57].

### Immunohistochemical Protein Assay

 Striatal tissue slices with a thickness of 4 µm were sectioned from paraffin blocks for immunohistochemical staining according to the manufacturer’s technique. Slices were subjected to treatment with 0.3% H2O2 for 20 min, followed by 10% normal blocking serum for 60 min at ambient temperature. Then, slices undergo an overnight incubation at 4 °C with anti-caspase-1 (1:50, ab138483, Abcam, MA, USA),anti-glial fibrillary acidic protein (GFAP, 1:1000, ab68428, Abcam, MA, USA) and anti-synaptophysin (1:100, ab14692, Abcam, MA, USA) primary antibodies. Then, the horseradish peroxidase (DAKO, CA, USA) was applied then treated for 15 min with diaminobenzidine (DAB). Slices were then subjected to dehydration and clearing in xylene, counter-stained with hematoxylin, and viewed under a microscope. After each step, tissue sections were rinsed with phosphate buffer saline [58]. To determine the area percentage of caspase-1, GFAP and synaptophysin immunoreactivity, a five randomly chosen separate fields (× 400) were chosen and photographed from striatal tissue from every tissue slice of each specimen then analysed using ImageJ analysis software (NIH, USA) [59].

###  Statistical Analysis

 Values were expressed as mean ± SE, and the Tukey–Kramer post-hoc test was used after analysis of variance (ANOVA) for group comparisons. After the Kruskal–Wallis test, Dunn’s test was used to examine non-parametric lesions. A significance level of *p* < 0.05 was used (GraphPad® program Inc., Version 10.3.1, San Diego, CA, USA). The Prism program was used for all statistical analyses [60].

## Results

### Phytochemical Characterization of the Methanolic Extract of *Vitex* Leaves

**Fig. 1:**
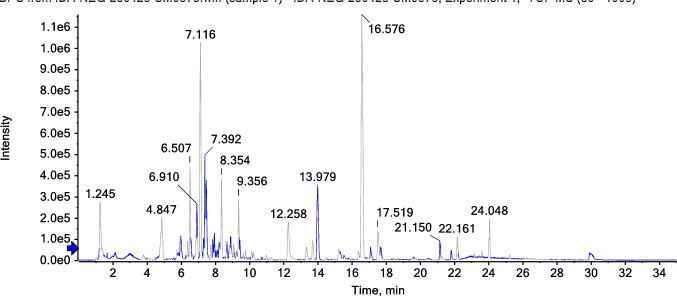
Total ion chromatogram of LC–ESI–MS/MS analysis (negative mode) of *Vitex* leaves extract

In the methanolic extract of *Vitex* leaves, 46 components were discovered in a provisional manner using the negative mode of LC-electrospray ionization (ESI)-MS/MS. The main components have different chemical ontologies, including iridoid, organic acids, flavonoids, coumarins, terpenoids, fatty acids, and other glycosylated compounds. The obtained metabolite profile is presented in Table 2 and Fig. 1.

**Table 2:**
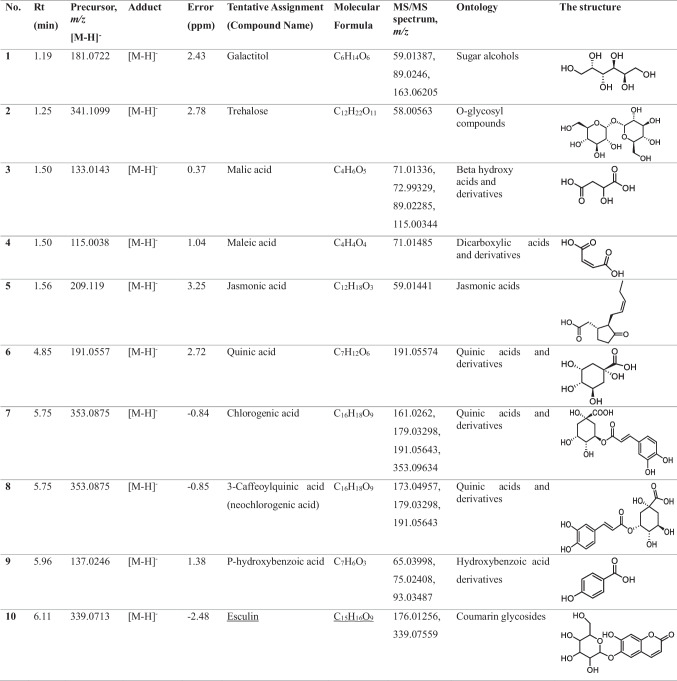

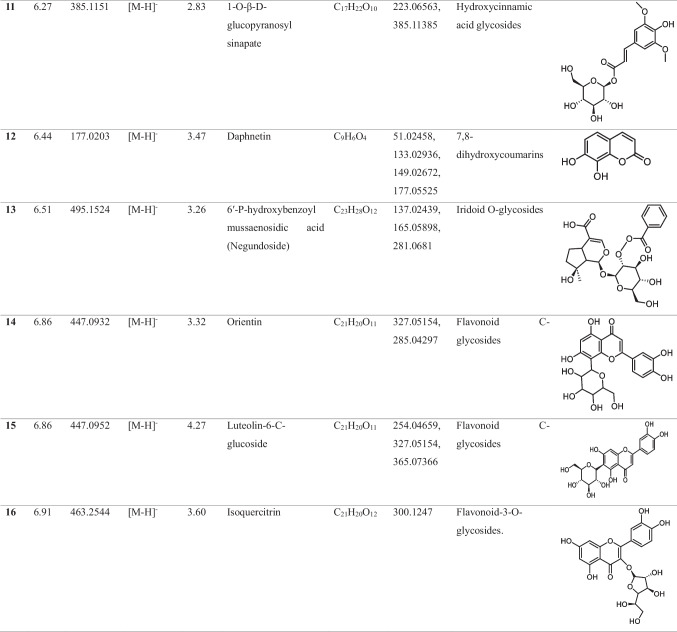

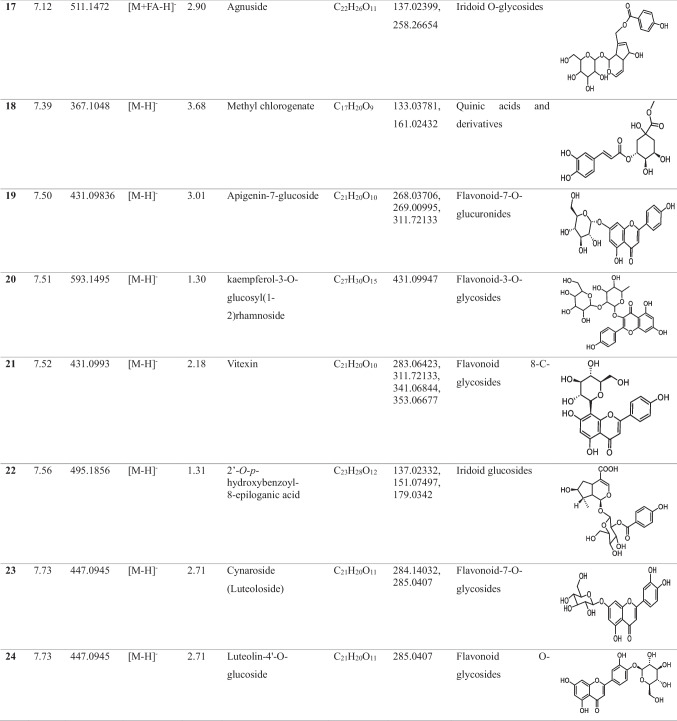

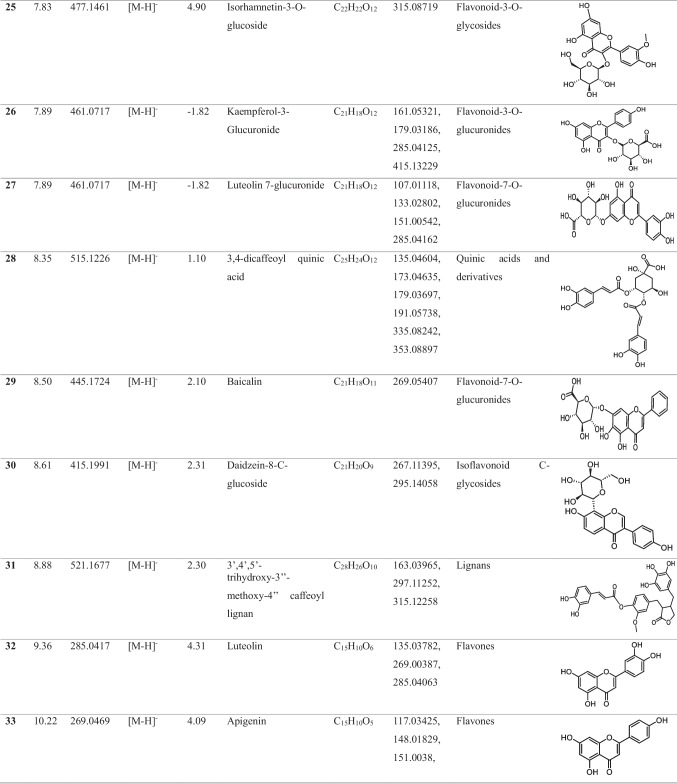

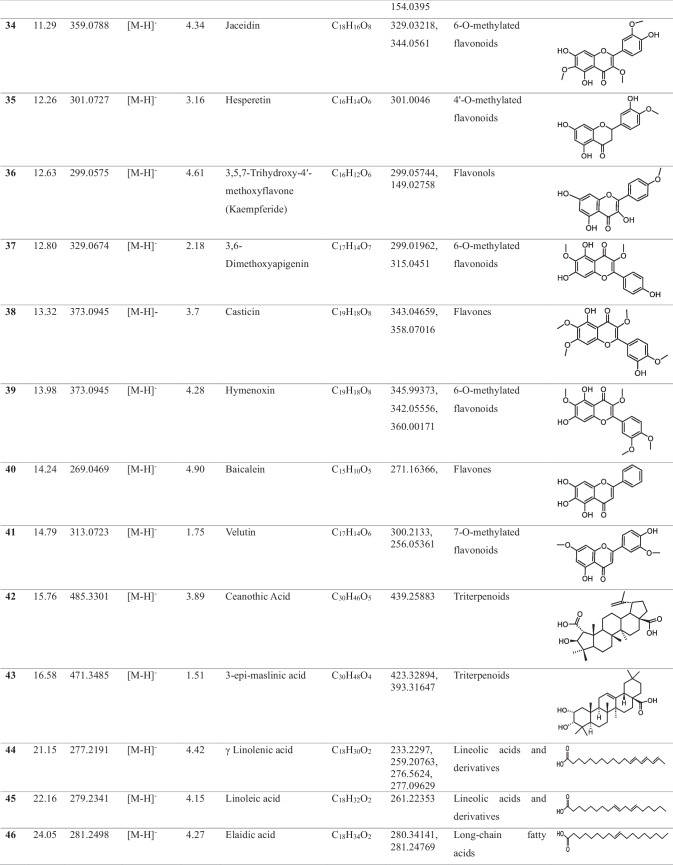
Natural compounds detected in *Vitex* leaf extract, using negative mode ionization LC-HRESI-MS/MS analysis

*LC–ESI–MS/MS* liquid chromatography-electrospray ionization-tandem mass spectrometry, *RT* retention time, *m/z* mass-to-charge ratio. ppm, 10^−6^

### Molecular Docking

**Fig. 2:**
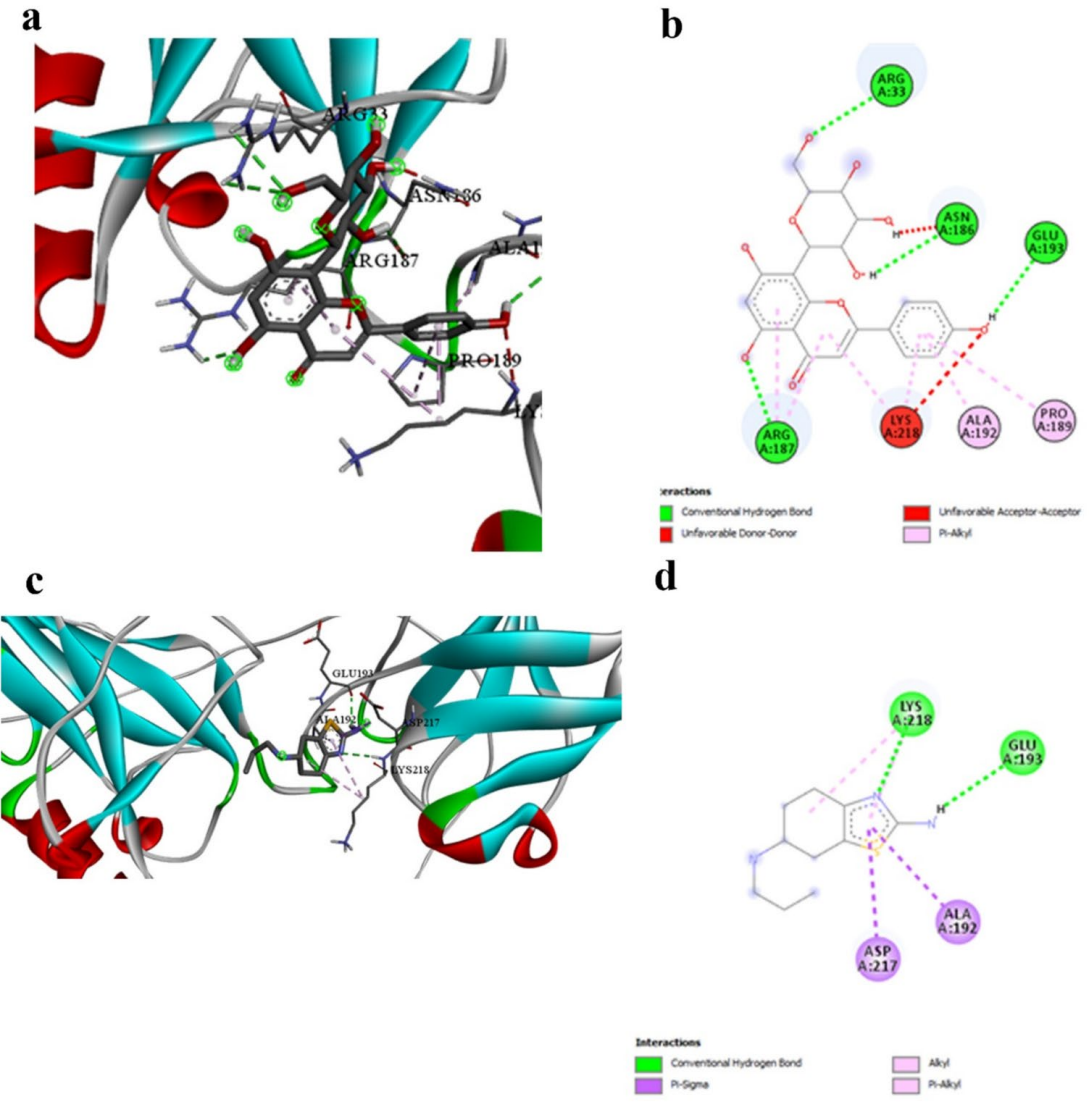
NFκB-p65 binding interactions. a and b 3D and 2D representations of Vitexin binding to NFκB-p65, showing engagement with DNA-binding residues ARG33 and ARG187. c and d 3D and 2D representations of pramipexole binding, showing peripheral interactions. Vitexin’s superior binding suggests enhanced potential for inhibiting transcriptional activity

Vitexin demonstrates superior binding with NFκB-p65 (− 6.8 kcal/mol, Table 3) compared to pramipexole (− 4.5 kcal/mol, Table 3), representing a 51% improvement in binding energy. This significant difference underscores the enhanced anti-inflammatory potential of natural compounds compared to conventional dopaminergic therapies. The interaction mechanisms reveal distinct targeting strategies (Table 4 vs. Table 11). Prami engages LYS218 and GLU193 through limited hydrogen bonding and establishes hydrophobic contacts with LYS218, ALA192, and ASP217. Vitexin, however, creates multiple hydrogen bonds with critical arginine residues (ARG33, ARG187) known to be involved in DNA binding, along with extensive hydrophobic π-alkyl interactions (Fig. 2a and b vs. Figure 2c and d). Vitexin’s engagement of DNA-binding residues suggests superior potential for inhibiting NFκB’s transcriptional activity compared to Prami’s more peripheral interactions. This enhanced anti-inflammatory capability could provide superior modulation of pro-inflammatory gene expression, offering improved therapeutic outcomes in neuroinflammatory conditions.

**Table 3:** Molecular docking binding affinity Δ*G* (kcal/mol) for each target protein and tested ligand in addition to the reference compound pramipexole. More negative values indicate a stronger binding affinity

Receptor	Ligand	Score	Prami score	%
NFKB_p65-Q7TQN4	Agnuside	− 6.1	− 4.5	35.55
	Casticin	− 5.5		22.22
	Luteolin-6-C-glucoside	− 6.6		46.66
	Orientin	− 6.6		46.66
	Vitexin	− 6.8		51.11
NLRP3-D4A523	Agnuside	− 9.5	− 6.3	50.79
	Casticin	− 7.8		23.80
	Luteolin-6-C-glucoside	− 8.1		28.57
	Orientin	− 9		42.85
	Vitexin	− 8.6		36.50
Casp1-P43527	Agnuside	− 6.7	− 4.6	45.65
	Casticin	− 5.7		23.91
	Luteolin-6-C-glucoside	− 6.6		43.47
	Orientin	− 6.9		50
	Vitexin	− 6.9		50
GSDMD-A0A096MJ11	Agnuside	− 8.6	− 5.5	56.36
	Casticin	− 7.3		32.72
	Luteolin-6-C-glucoside	− 8.5		54.54
	Orientin	− 7.7		40
	Vitexin	− 8		45.45
Alpha-synuclein-P37377	Agnuside	− 5	− 3.4	47.05
	Casticin	− 4.6		35.29
	Luteolin-6-C-glucoside	− 4.9		44.11
	Orientin	− 5		47.05
	Vitexin	− 5.3		55.88
Tyrosine_hydroxylase-P04177	Agnuside	− 8.2	− 6.8	20.58
	Casticin	− 6.9		1.47
	Luteolin-6-C-glucoside	− 8.4		23.52
	Orientin	− 7.8		14.70
	Vitexin	− 7.9		16.17
Dopamin_receptor-P30729	Agnuside	− 9.3	− 6.2	50
	Casticin	− 7.6		22.58
	Luteolin-6-C-glucoside	− 8.7		40.32
	Orientin	− 7.8		25.80
	Vitexin	− 8.7		40.32

**Table 4:** Interaction profile between NFκB-p65 and the best-performing ligand Vitexin, demonstrating engagement with DNA-binding residues critical for transcriptional regulation

Interaction	Distance	Category	Type
A:ARG33:HH11 -:LIGAND1:O	2.86009	Hydrogen bond	Conventional hydrogen bond
A:ARG33:HH21 -:LIGAND1:O	3.03661	Hydrogen bond	Conventional hydrogen bond
A:ARG187:HH21 -:LIGAND1:O	2.38727	Hydrogen bond	Conventional hydrogen bond
:LIGAND1:H—A:ASN186:O	2.8785	Hydrogen bond	Conventional hydrogen bond
:LIGAND1:H—A:GLU193:O	3.05965	Hydrogen bond	Conventional hydrogen bond
:LIGAND1—A:ARG187	4.2317	Hydrophobic	Pi-alkyl
:LIGAND1—A:LYS218	5.48273	Hydrophobic	Pi-alkyl
:LIGAND1—A:ARG187	4.55294	Hydrophobic	Pi-alkyl
:LIGAND1—A:PRO189	5.376	Hydrophobic	Pi-alkyl
:LIGAND1—A:ALA192	4.20035	Hydrophobic	Pi-alkyl
:LIGAND1—A:LYS218	3.73137	Hydrophobic	Pi-alkyl

**Fig. 3:**
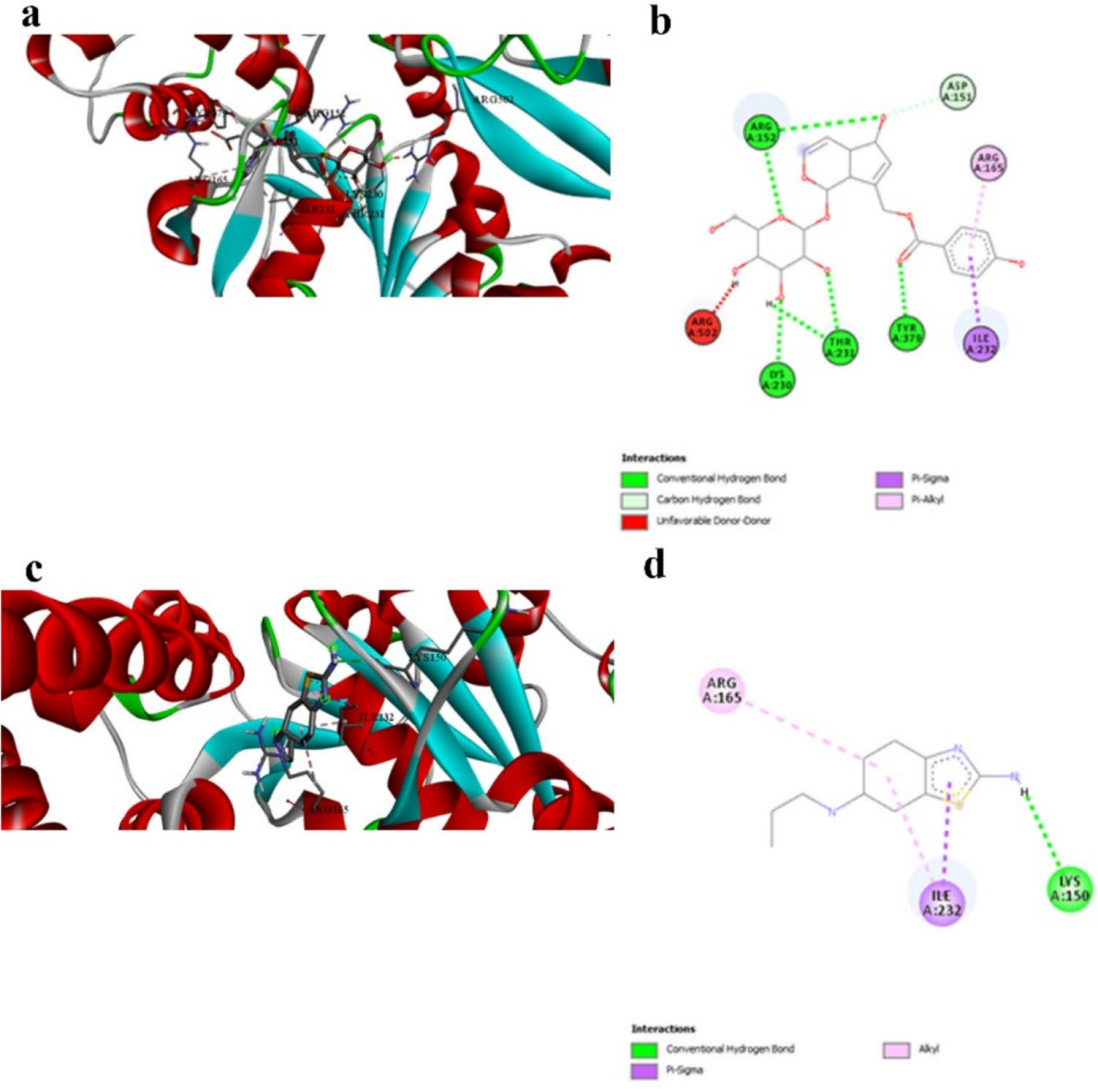
NLRP3 binding interactions. **a** and **b** 3D and 2D representations of Agnuside binding to NLRP3, demonstrating the most extensive interaction network in this study, including critical engagement with TYR379. **c** and **d** 3D and 2D representations of pramipexole binding, showing limited interactions. The exceptional binding affinity of Agnuside (− 9.5 kcal/mol) represents the strongest interaction observed across all targets

Agnuside exhibits the strongest binding affinity observed in this study with NLRP3 (− 9.5 kcal/mol, Table 3), dramatically exceeding Prami’s interaction (− 6.3 kcal/mol, Table 3) by 51%. This represents the most significant difference observed across all targets, highlighting Agnuside’s exceptional anti-inflammatory potential. Prami’s interaction with NLRP3 is limited to hydrogen bonding with LYS150 and hydrophobic interactions with ILE232 and ARG165 (Table 11). Agnuside establishes a comprehensive interaction network involving seven distinct molecular interactions, including hydrogen bonds with ARG152, LYS230, THR231, and the critical TYR379 (Table 5 and Fig. 3a and b vs. Figure 3c and d). The engagement of TYR379 by Agnuside is particularly significant, as tyrosine phosphorylation regulates NLRP3 inflammasome activation. This strategic interaction, absent in Prami, suggests superior potential for inflammasome inhibition. The extensive binding network positions Agnuside as a highly effective NLRP3 inhibitor with applications extending far beyond traditional dopaminergic therapy.

**Table 5:** Comprehensive interaction analysis between NLRP3 and the best-performing ligand Agnuside, showing the most extensive binding network observed in this study

Interaction	Distance	Category	Type
A:ARG152:HN -:LIGAND1:O	2.64633	Hydrogen bond	Conventional hydrogen bond
A:ARG152:HH22 -:LIGAND1:O	2.13059	Hydrogen bond	Conventional hydrogen bond
A:LYS230:HN -:LIGAND1:O	2.68913	Hydrogen bond	Conventional hydrogen bond
A:THR231:HN -:LIGAND1:O	2.59866	Hydrogen bond	Conventional hydrogen bond
A:TYR379:HH -:LIGAND1:O	2.33755	Hydrogen bond	Conventional hydrogen bond
:LIGAND1:H—A:THR231:OG1	2.97957	Hydrogen bond	Conventional hydrogen bond
:LIGAND1:H -:LIGAND1:O	2.9557	Hydrogen bond	Conventional hydrogen bond
A:ASP151:CA -:LIGAND1:O	3.40995	Hydrogen bond	Carbon hydrogen bond
A:ILE232:CG2 -:LIGAND1	3.85143	Hydrophobic	Pi-sigma
:LIGAND1—A:ARG165	4.83943	Hydrophobic	Pi-alkyl

**Fig. 4:**
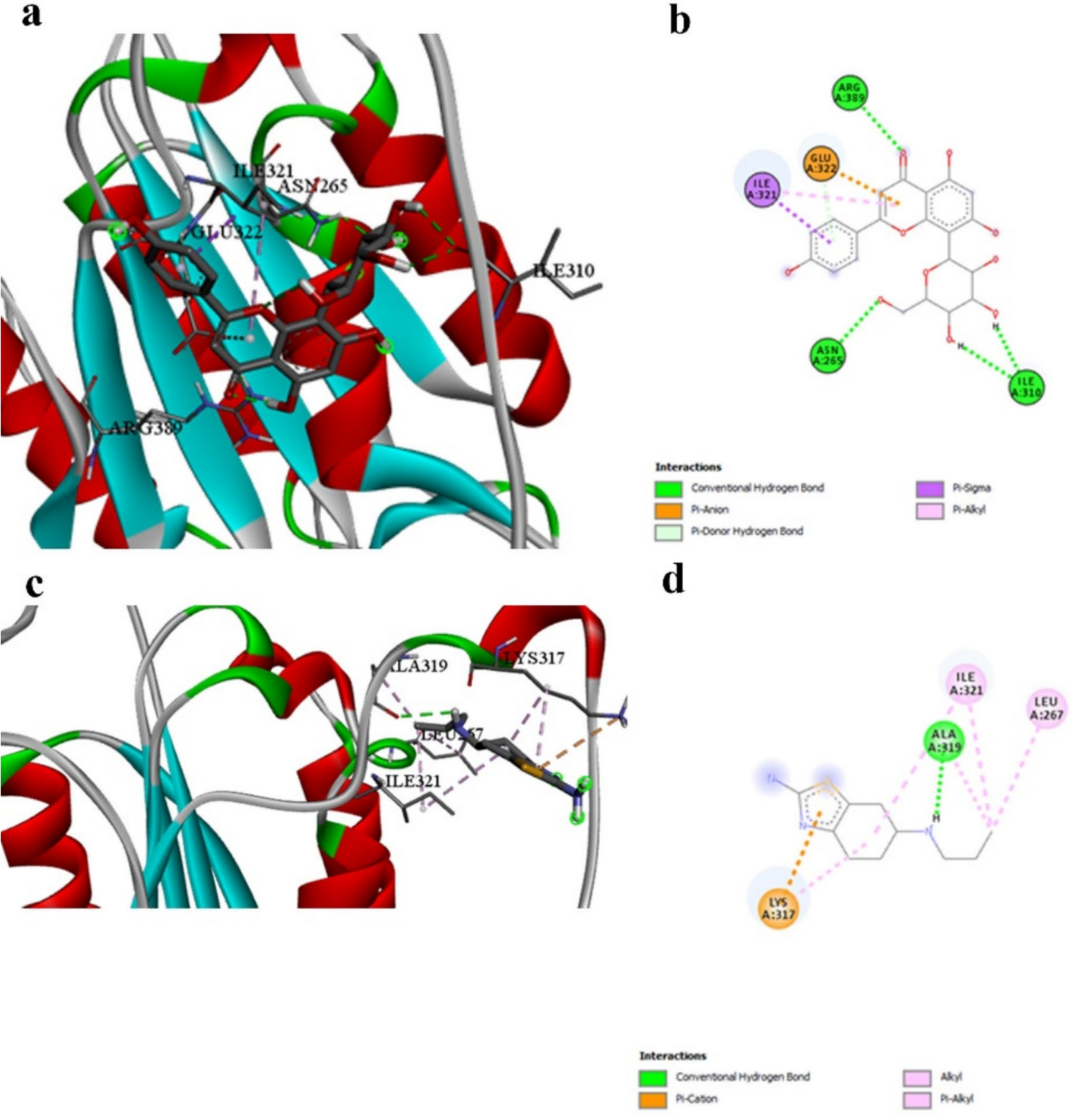
Caspase-1 binding interactions. **a** and **b** 3D and 2D representations of Vitexin binding to Caspase-1, showing extensive hydrogen bonding network with ASN265, ARG389, and ILE310, plus electrostatic interactions with GLU322. **c** and **d** 3D and 2D representations of pramipexole binding, showing limited hydrogen bonding with ALA319. The 50% improvement in binding affinity for Vitexin reflects its more comprehensive interaction profile

The inflammatory targeting capability of our test compounds dramatically exceeds that of Prami (Table 3). Both Vitexin and Orientin demonstrate exceptional binding affinities with caspase-1 (− 6.9 kcal/mol each), compared to Prami’s moderate affinity (− 4.6 kcal/mol, Table 3). This represents a 50% improvement in binding energy, suggesting significantly enhanced anti-inflammatory potential. Prami’s interaction with caspase-1 is limited to hydrogen bonding with ALA319 and various hydrophobic interactions with LYS317, ILE321, and LEU267 (Table 11). In contrast, Vitexin establishes an elaborate network of six conventional hydrogen bonds with multiple residues (ASN265, ARG389, ILE310) and additional electrostatic interactions with GLU322 (Table 6 and Fig. 4a and b). This more extensive interaction profile suggests superior inhibitory potential against caspase-1 activity compared to the reference compound shown in Fig. 4c and d. The strategic engagement of ASN265 and ARG389 by Vitexin, residues proximal to the catalytic site, contrasts with Prami’s interaction with the more peripheral ALA319. This positional advantage, combined with the superior binding energy, positions Vitexin as a more effective caspase-1 inhibitor with enhanced anti-inflammatory capabilities relevant to neurological disorders.

**Table 6:** Comprehensive interaction profile between caspase-1 and the best-performing ligand Vitexin, demonstrating multiple hydrogen bonds and hydrophobic interactions that contribute to superior binding affinity

Interaction	Distance	Category	Type
A:ASN265:HD21 -:LIGAND1:O	2.2399	Hydrogen bond	Conventional hydrogen bond
A:ARG389:HH21 -:LIGAND1:O	2.70035	Hydrogen bond	Conventional hydrogen bond
:LIGAND1:H—A:ILE310:O	2.76728	Hydrogen bond	Conventional hydrogen bond
:LIGAND1:H—A:ILE310:O	2.1263	Hydrogen bond	Conventional hydrogen bond
:LIGAND1:H -:LIGAND1:O	3.00574	Hydrogen bond	Conventional hydrogen bond
:LIGAND1:H -:LIGAND1:O	2.18167	Hydrogen bond	Conventional hydrogen bond
A:GLU322:OE2 -:LIGAND1	4.08314	Electrostatic	Pi-anion
A:GLU322:HN -:LIGAND1	3.02932	Hydrogen bond	Pi-donor hydrogen bond
A:ILE321:CB -:LIGAND1	3.73614	Hydrophobic	Pi-sigma
:LIGAND1—A:ILE321	5.31754	Hydrophobic	Pi-alkyl

**Fig. 5:**
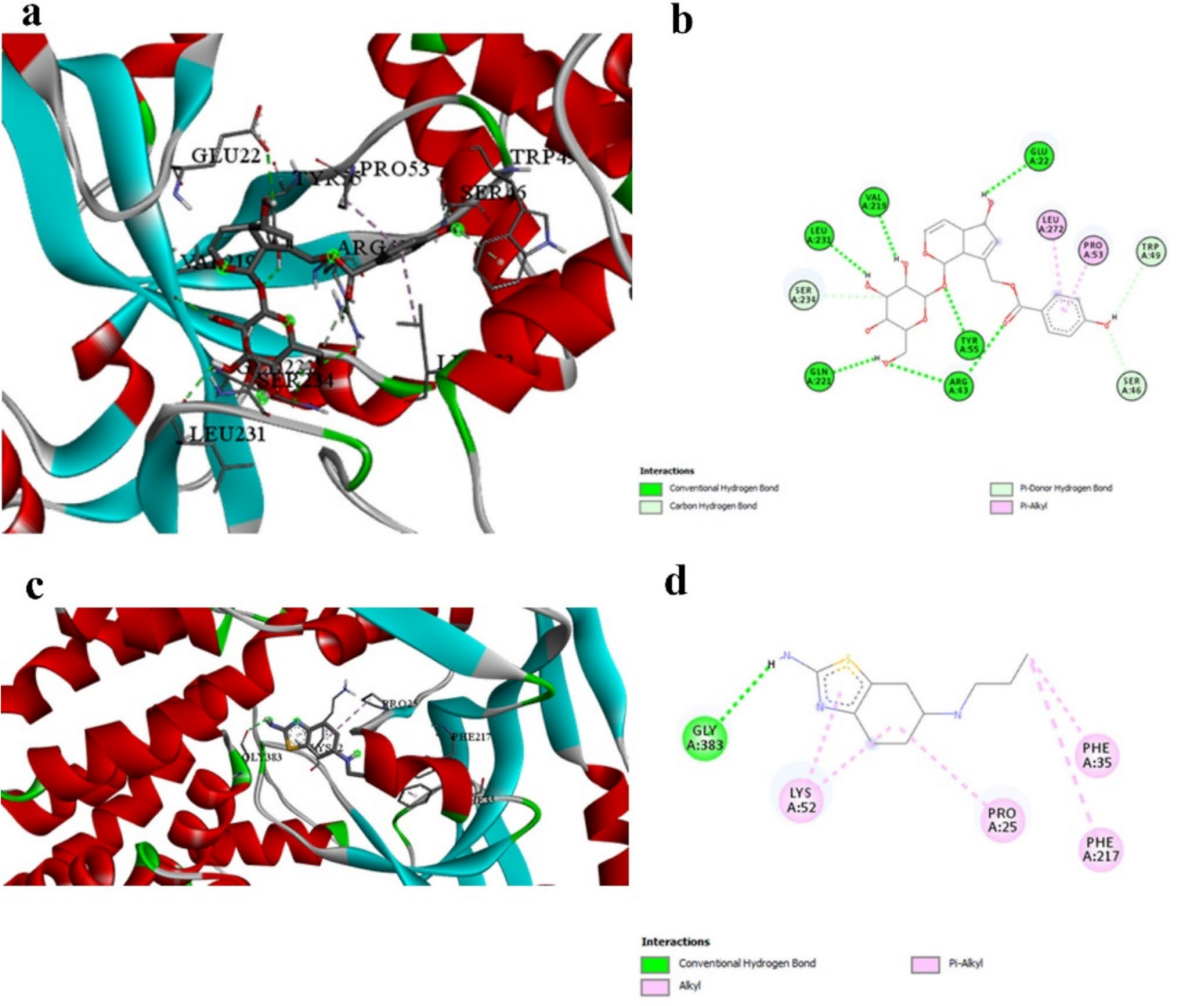
GSDMD binding interactions. **a** and **b** 3D and 2D representations of Agnuside binding to GSDMD, showing an extensive hydrogen bonding network with seven conventional hydrogen bonds. **c** and **d** 3D and 2D representations of pramipexole binding, demonstrating limited interaction. The 56% improvement in binding affinity for Agnuside highlights its potential for addressing pyroptotic cell death mechanisms

Gasdermin D represents a target not traditionally addressed by current PD therapeutics. Agnuside demonstrates strong binding affinity with GSDMD (− 8.6 kcal/mol, Table 3), substantially exceeding Prami’s moderate interaction (− 5.5 kcal/mol, Table 3) by 56%. This difference highlights the potential of natural compounds to address pyroptotic cell death mechanisms not targeted by conventional therapies. Prami’s interaction with GSDMD is limited to hydrogen bonding with GLY383 and hydrophobic interactions with PRO25, LYS52, PHE35, and PHE217 (Table 11). In contrast, Agnuside establishes an extensive network of seven conventional hydrogen bonds with multiple critical residues (ARG43, TYR55, GLN221, LEU231, VAL219, GLU22) and additional carbon-hydrogen bonds (Table 7 and Fig. 5a and b vs. Figure 5c and d). The superior binding profile of Agnuside suggests enhanced potential for GSDMD inhibition, which could translate to improved neuroprotection through reduced pyroptotic cell death. This mechanism represents a novel therapeutic approach that complements traditional dopaminergic strategies, potentially offering enhanced neuroprotective effects in neurodegenerative disorders.

**Table 7:** Detailed binding interactions between GSDMD and the best-performing ligand Agnuside, showing an extensive hydrogen bonding network contributing to strong binding affinity

Interaction	Distance	Category	Type
A:ARG43:HH11 -:LIGAND1:O	2.58852	Hydrogen bond	Conventional hydrogen bond
A:ARG43:HH21 -:LIGAND1:O	2.56968	Hydrogen bond	Conventional hydrogen bond
A:TYR55:HH -:LIGAND1:O	2.68092	Hydrogen bond	Conventional hydrogen bond
:LIGAND1:H—A:GLN221:OE1	2.44251	Hydrogen bond	Conventional hydrogen bond
:LIGAND1:H—A:LEU231:O	2.5857	Hydrogen bond	Conventional hydrogen bond
:LIGAND1:H—A:VAL219:O	2.21283	Hydrogen bond	Conventional hydrogen bond
:LIGAND1:H—A:GLU22:OE2	2.78813	Hydrogen bond	Conventional hydrogen bond
A:SER46:CB -:LIGAND1:O	3.61043	Hydrogen bond	Carbon hydrogen bond
:LIGAND1:C—A:SER234:OG	2.98817	Hydrogen bond	Carbon hydrogen bond
:LIGAND1:C -:LIGAND1:O	3.67127	Hydrogen bond	Carbon hydrogen bond
:LIGAND1:H—A:TRP49	3.20736	Hydrogen bond	Pi-donor hydrogen bond
:LIGAND1—A:PRO53	4.68273	Hydrophobic	Pi-alkyl
:LIGAND1—A:LEU272	4.85534	Hydrophobic	Pi-alkyl

**Fig. 6:**
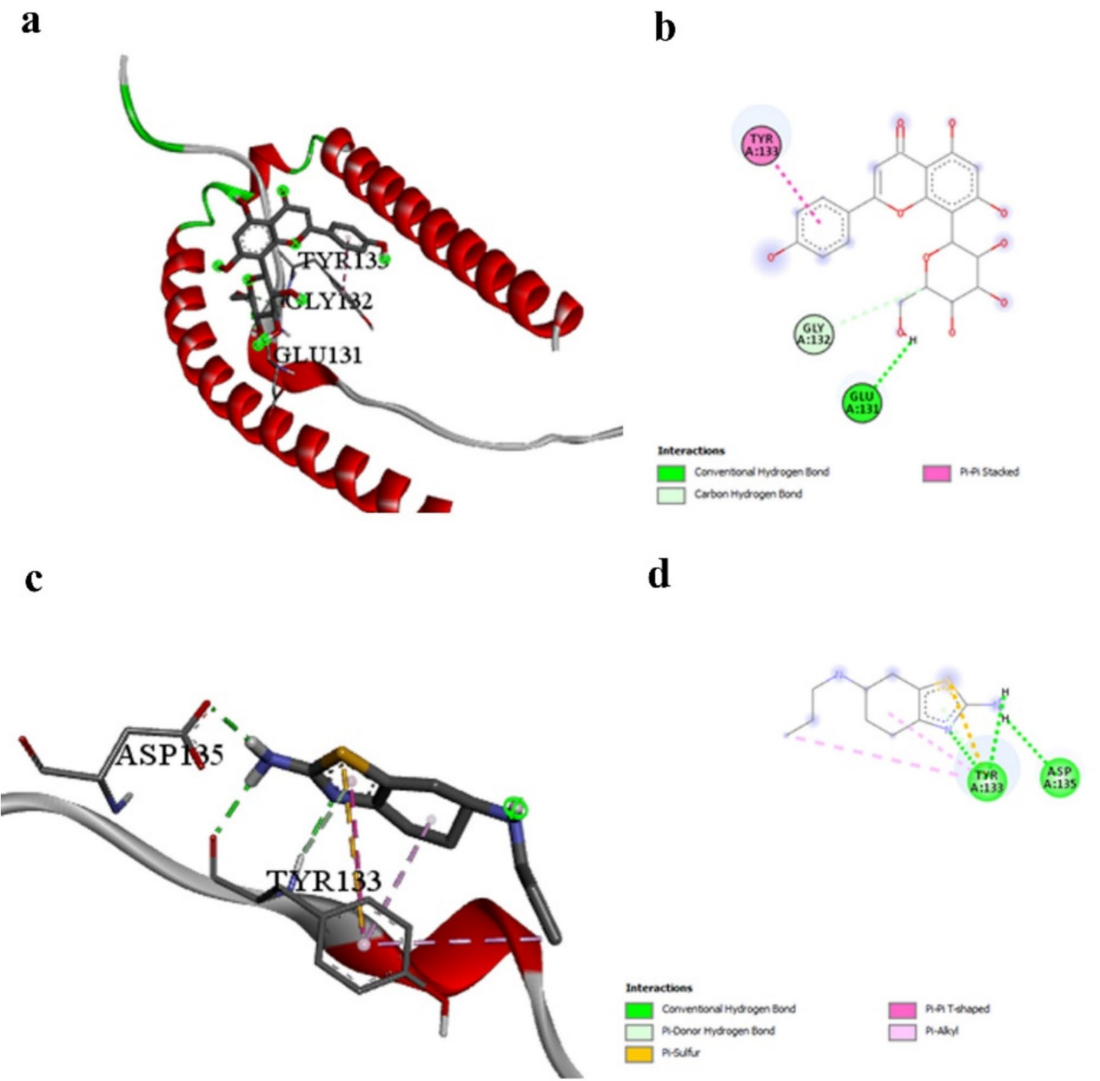
Alpha-synuclein binding interactions. **a** and **b** 3D and 2D representations of Vitexin binding to Alpha-synuclein, showing π-π stacking with TYR133 and hydrogen bonding with GLU131. **c** and **d** 3D and 2D representations of pramipexole (reference) binding, demonstrating hydrogen bonding with TYR133 and ASP135. The superior binding affinity of Vitexin (− 5.3 kcal/mol) compared to pramipexole (− 3.4 kcal/mol) is evident in the more extensive interaction network

Our docking results (Table 3) indicate that Vitexin exhibits significantly stronger binding affinity (− 5.3 kcal/mol) with α-synuclein compared to Prami (− 3.4 kcal/mol, Table 3), representing a 56% improvement in binding energy. This substantial difference suggests that Vitexin may offer superior therapeutic potential for targeting α-synuclein pathology compared to the current clinical standard. The interaction profiles reveal distinct binding mechanisms between the compounds (Tables 8 and 11). While Prami demonstrates conventional hydrogen bonding with TYR133 and ASP135, along with hydrophobic π-π interactions, Vitexin engages different residues through conventional hydrogen bonding with GLU131 and π-π stacking with TYR133 (Fig. 6a and b vs. Figure 6c and d). Notably, both compounds interact with TYR133, but through different mechanisms—Prami via hydrogen bonding and Vitexin via π-π stacking. This convergence on a common residue validates TYR133 as a critical binding site, while the different interaction modes suggest complementary mechanisms that could potentially be exploited therapeutically. The π-π stacking interaction of Vitexin with TYR133 is particularly significant, as tyrosine residues in α-synuclein have been implicated in protein aggregation through oxidative coupling mechanisms. This interaction mode may provide superior interference with aggregation-promoting conformational changes compared to the hydrogen bonding observed with Prami, potentially explaining the enhanced binding affinity.

**Table 8:** Detailed interaction analysis between Alpha-synuclein and the best-performing ligand Vitexin, showing hydrogen bonding distances, interaction categories, and specific binding types

Interaction	Distance	Category	Type
:LIGAND1:H—A:GLU131:O	2.20126	Hydrogen bond	Conventional hydrogen bond
:LIGAND1:C—A:GLY132:O	3.15414	Hydrogen bond	Carbon hydrogen bond
A:TYR133 -:LIGAND1	4.54122	Hydrophobic	Pi-Pi stacked

**Fig. 7:**
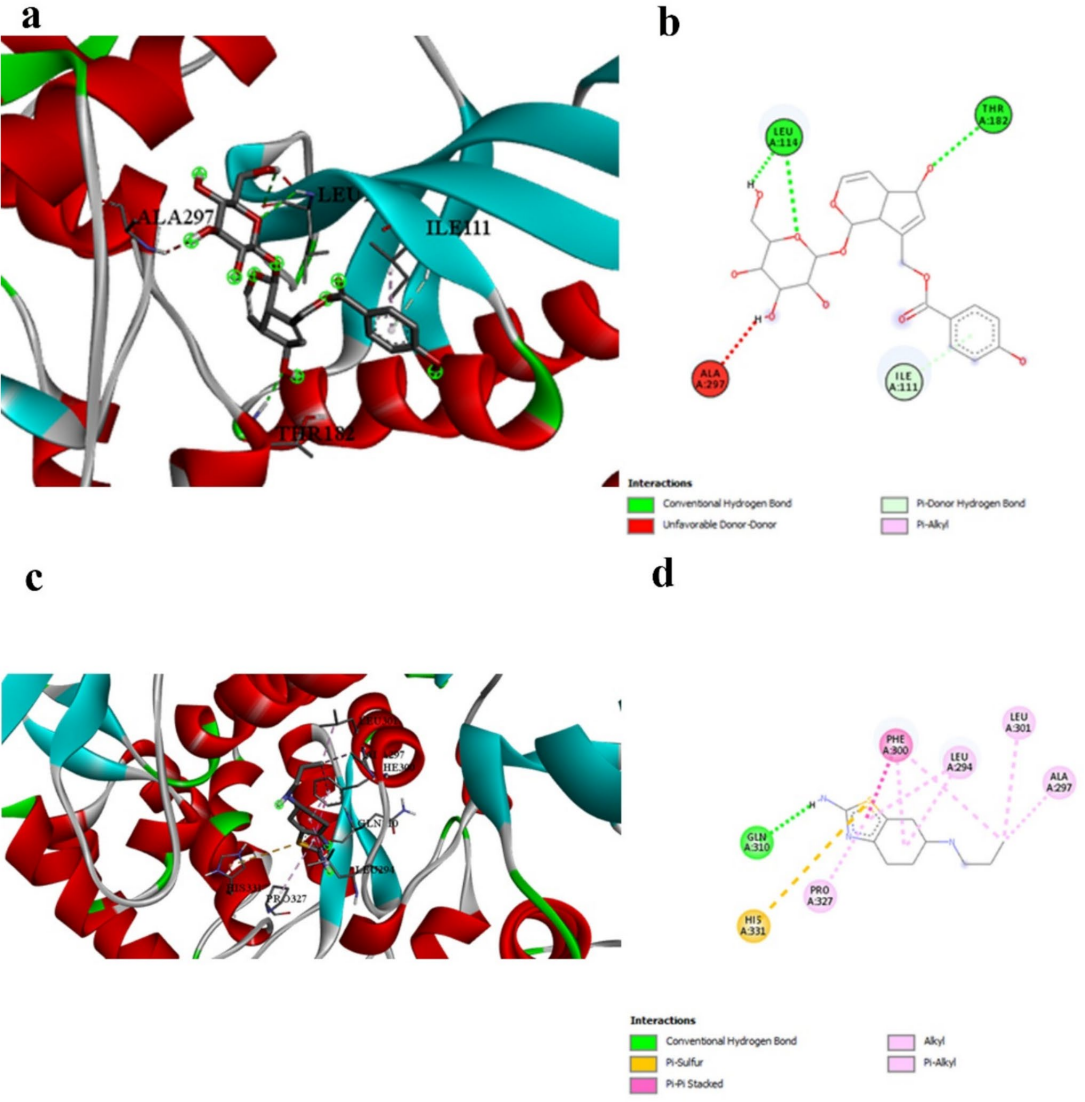
Tyrosine hydroxylase binding interactions. **a** and **b** 3D and 2D representations of Agnuside binding to tyrosine hydroxylase, showing hydrogen bonding with LEU114 and THR182. **c** and **d** 3D and 2D representations of pramipexole binding, demonstrating different binding site engagement. Both compounds show complementary binding mechanisms, suggesting potential for synergistic effects on catecholamine synthesis

Agnuside demonstrates superior binding with tyrosine hydroxylase (− 8.2 kcal/mol, Table 3) compared to Prami (− 6.8 kcal/mol, Table 3), representing a 21% improvement. While this difference is smaller than observed with other targets, it remains clinically relevant given tyrosine hydroxylase’s central role in dopamine synthesis. The interaction profiles reveal complementary mechanisms (Table 9 vs. Table 11). Prami engages GLN310 through hydrogen bonding and establishes extensive hydrophobic interactions with PHE300, ALA297, LEU294, LEU301, and PRO327. Agnuside creates hydrogen bonds with LEU114 and THR182, along with π-donor hydrogen bonding with ILE111 and hydrophobic interaction with ILE111 (Fig. 7a and b vs. Figure 7c and d).

**Table 9:** Interaction details between tyrosine hydroxylase and the best-performing ligand Agnuside, illustrating complementary binding mechanisms compared to the reference compound

Interaction	Distance	Category	Type
A:LEU114:HN -:LIGAND1:O	2.92712	Hydrogen bond	Conventional hydrogen bond
A:THR182:HN -:LIGAND1:O	2.37365	Hydrogen bond	Conventional hydrogen bond
:LIGAND1:H—A:LEU114:O	3.01658	Hydrogen bond	Conventional hydrogen bond
A:ILE111:HN -:LIGAND1	3.04649	Hydrogen bond	Pi-donor hydrogen bond
:LIGAND1—A:ILE111	4.23607	Hydrophobic	Pi-alkyl

**Fig. 8:**
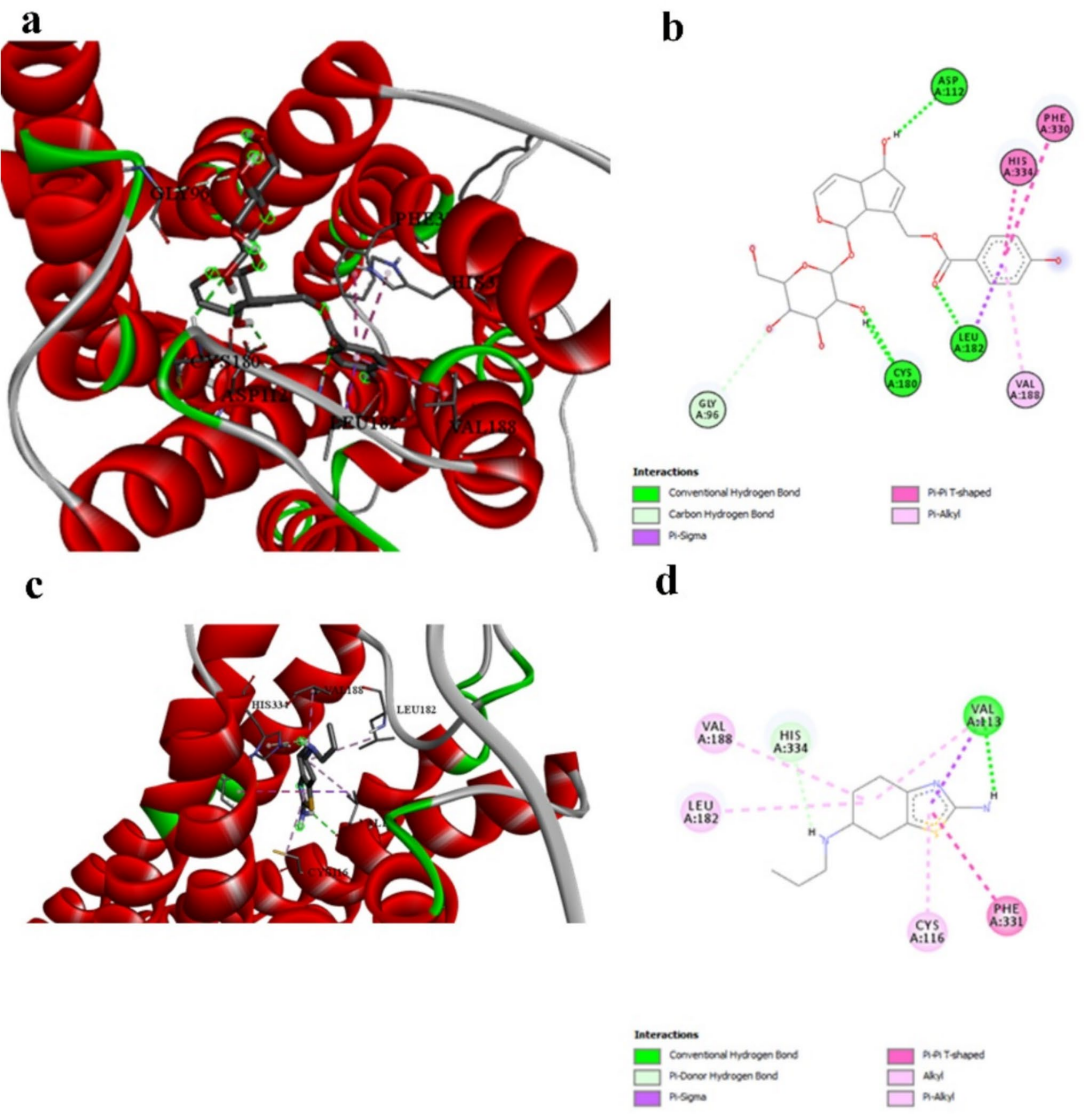
Dopamine receptor binding interactions. **a** and **b** 3D and 2D representations of Agnuside binding to dopamine receptor, highlighting critical hydrogen bonding with ASP112 and extensive hydrophobic interactions. **c** and **d** 3D and 2D representations of pramipexole binding, showing different binding site engagement. Agnuside’s superior affinity (− 9.3 kcal/mol) compared to pramipexole (− 6.2 kcal/mol) demonstrates enhanced dopaminergic targeting potential

Agnuside demonstrates remarkable binding affinity with the dopamine receptor (− 9.3 kcal/mol, Table 3), substantially exceeding pramipexole’s performance (− 6.2 kcal/mol, Table 3) by 50%. This exceptional difference is particularly significant given that dopamine receptor modulation is Prami’s primary therapeutic mechanism in clinical practice. The interaction profiles reveal complementary but distinct binding mechanisms (Table 10 vs. Table 11). Pramipexole engages VAL113 and HIS334 through hydrogen bonding and establishes hydrophobic contacts with multiple residues, including PHE331, VAL188, LEU182, and CYS116. Agnuside, however, creates a more extensive hydrogen bonding network with CYS180, LEU182, and the critical ASP112, while maintaining hydrophobic interactions with aromatic residues PHE330 and HIS334 (Fig. 8a and b vs. Figure 8c and d). The engagement of ASP112 by Agnuside is particularly noteworthy, as this aspartate residue is crucial for ligand recognition in G-protein-coupled receptors. Prami does not interact with this critical residue, potentially explaining Agnuside’s superior binding affinity. Furthermore, both compounds interact with aromatic residues (PHE330/PHE331 and HIS334), but Agnuside’s π-π T-shaped interactions may provide more stable binding compared to Prami’s simple hydrophobic contacts.

**Table 10:** Interaction analysis between dopamine receptor and the best-performing ligand Agnuside, highlighting critical hydrogen bonds with key residues and hydrophobic contacts

Interaction	Distance	Category	Type
A:CYS180:HN -:LIGAND1:O	2.74445	Hydrogen bond	Conventional hydrogen bond
A:LEU182:HN -:LIGAND1:O	2.07388	Hydrogen bond	Conventional hydrogen bond
:LIGAND1:H—A:CYS180:O	1.92813	Hydrogen bond	Conventional hydrogen bond
:LIGAND1:H—A:ASP112:OD2	2.09778	Hydrogen bond	Conventional hydrogen bond
A:GLY96:CA -:LIGAND1:O	3.64727	Hydrogen bond	Carbon hydrogen bond
A:LEU182:CD1 -:LIGAND1	3.45983	Hydrophobic	Pi-sigma
A:PHE330 -:LIGAND1	4.96518	Hydrophobic	Pi-Pi T-shaped
A:HIS334 -:LIGAND1	4.70308	Hydrophobic	Pi-Pi T-shaped
:LIGAND1—A:VAL188	5.29519	Hydrophobic	Pi-alkyl

**Table 11:** Complete interaction profiles of the reference compound (pramipexole) with all target proteins, providing a baseline comparison for evaluating natural compound performance

Protein	Ligand	Interaction	Distance	Category
NFKB	Reference	A:LYS218 -:UNL1	4.87498	Hydrophobic
	Reference	A:LYS218:HN -:UNL1:N	2.73474	Hydrogen bond
	Reference	:UNL1:H—A:GLU193:O	2.416	Hydrogen bond
	Reference	:UNL1—A:LYS218	4.83404	Hydrophobic
	Reference	A:ALA192:CB -:UNL1	3.71262	Hydrophobic
	Reference	A:ASP217:CB -:UNL1	3.97431	Hydrophobic
NLRP3	Reference	:UNL1:H—A:LYS150:O	2.68377	Hydrogen bond
	Reference	A:ILE232:CD1 -:UNL1	3.7665	Hydrophobic
	Reference	A:ARG165 -:UNL1	5.13775	Hydrophobic
	Reference	:UNL1—A:ILE232	3.95148	Hydrophobic
Caspase-1	Reference	:UNL1:H—A:ALA319:O	2.84667	Hydrogen bond
	Reference	A:LYS317:NZ -:UNL1	4.90496	Electrostatic
	Reference	A:LYS317 -:UNL1	3.78634	Hydrophobic
	Reference	A:ALA319 -:UNL1:C	4.07436	Hydrophobic
	Reference	:UNL1—A:ILE321	5.32198	Hydrophobic
	Reference	:UNL1:C—A:LEU267	4.93318	Hydrophobic
	Reference	:UNL1:C—A:ILE321	4.35731	Hydrophobic
	Reference	:UNL1—A:LYS317	4.01735	Hydrophobic
GSDMD	Reference	:UNL1:H—A:GLY383:O	2.56519	Hydrogen bond
	Reference	A:PRO25 -:UNL1	5.12426	Hydrophobic
	Reference	A:LYS52 -:UNL1	3.91686	Hydrophobic
	Reference	A:PHE35 -:UNL1:C	4.7407	Hydrophobic
	Reference	A:PHE217 -:UNL1:C	4.89532	Hydrophobic
	Reference	:UNL1—A:LYS52	4.43953	Hydrophobic
α-Synuclein	Reference	A:TYR133:HN -:UNL1:N	2.11954	Hydrogen bond
	Reference	:UNL1:H—A:TYR133:O	2.10479	Hydrogen bond
	Reference	:UNL1:H—A:ASP135:OD2	2.05455	Hydrogen bond
	Reference	A:TYR133:HN -:UNL1	2.84971	Hydrogen bond
	Reference	:UNL1:S—A:TYR133	5.57178	Other
	Reference	:UNL1—A:TYR133	5.22902	Hydrophobic
	Reference	A:TYR133 -:UNL1	4.80894	Hydrophobic
	Reference	A:TYR133 -:UNL1:C	5.38861	Hydrophobic
Tyrosine_hydroxylase	Reference	:UNL1:H—A:GLN310:O	2.30961	Hydrogen bond
	Reference	:UNL1:S—A:HIS331	5.91315	Other
	Reference	:UNL1—A:PHE300	4.20869	Hydrophobic
	Reference	A:ALA297 -:UNL1:C	3.64669	Hydrophobic
	Reference	:UNL1—A:LEU294	4.33599	Hydrophobic
	Reference	:UNL1:C—A:LEU301	3.91268	Hydrophobic
	Reference	A:PHE300 -:UNL1	3.98885	Hydrophobic
	Reference	A:PHE300 -:UNL1:C	4.78662	Hydrophobic
	Reference	:UNL1—A:LEU294	4.98464	Hydrophobic
	Reference	:UNL1—A:PRO327	4.84559	Hydrophobic
Dopamine	Reference	:UNL1:H—A:VAL113:O	2.592	Hydrogen bond
	Reference	:UNL1:H—A:HIS334	3.0547	Hydrogen bond
	Reference	A:VAL113:CG2 -:UNL1	3.5683	Hydrophobic
	Reference	:UNL1—A:PHE331	4.93253	Hydrophobic
	Reference	A:VAL113 -:UNL1	5.10817	Hydrophobic
	Reference	A:VAL188 -:UNL1	4.98934	Hydrophobic
	Reference	:UNL1—A:LEU182	5.27201	Hydrophobic
	Reference	:UNL1—A:CYS116	4.9177	Hydrophobic

Both compounds target the enzyme through distinct binding sites, suggesting potential for complementary therapeutic effects. Agnuside’s engagement of different residues may provide alternative modulation of catalytic activity, potentially offering enhanced or more sustained effects on catecholamine synthesis compared to pramipexole alone.

The comprehensive comparison with Prami (Table 3) reveals that the natural compounds consistently demonstrate superior or complementary binding profiles across all tested targets. Most significantly, several compounds exceed pramipexole’s performance by 50% or more in binding affinity, suggesting substantially enhanced therapeutic potential.

Agnuside emerges as particularly promising, demonstrating superior binding compared to pramipexole across five of seven targets, with exceptional performance in dopamine receptor (− 9.3 vs − 6.2 kcal/mol), NLRP3 (− 9.5 vs − 6.3 kcal/mol), and GSDMD (− 8.6 vs − 5.5 kcal/mol) interactions. This multi-target superiority suggests potential for addressing the complex pathophysiology of neurological disorders more comprehensively than current monotherapy approaches.

Vitexin complements this profile with superior performance in α-synuclein (− 5.3 vs − 3.4 kcal/mol), caspase-1 (− 6.9 vs − 4.6 kcal/mol), and NF-κB p65 (− 6.8 vs − 4.5 kcal/mol) targeting. The combination of Agnuside’s dopaminergic and anti-inflammatory effects with Vitexin’s anti-aggregation and anti-inflammatory properties could provide synergistic therapeutic benefits.

The structural basis for the enhanced binding affinities lies in the compounds’ ability to form multiple hydrogen bonds through their hydroxyl groups, engage in π-π interactions with aromatic residues, while their glycosidic moieties engage in additional hydrogen bonding interactions.

### ADMET Results

**Fig. 9:**
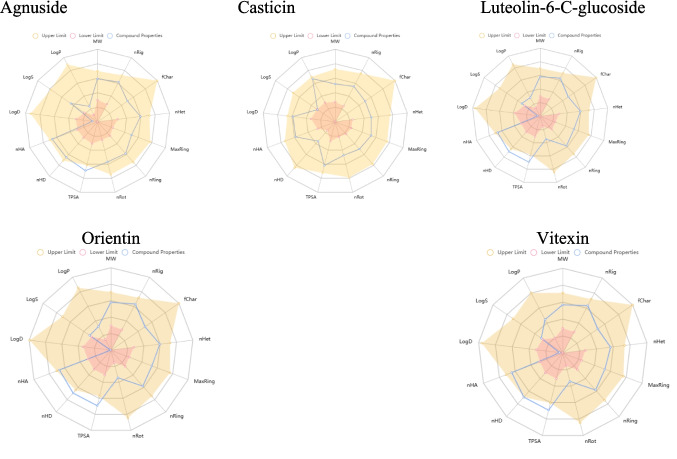
ADMET results for selected flavonoids

The ADMET (Absorption, Distribution, Metabolism, Excretion, and Toxicity) analysis provides crucial insights into the drug-like properties and potential clinical viability of the five Vitex compounds (Agnuside, Casticin, Luteolin-6-C-glucoside, Orientin, and Vitexin) identified through molecular docking studies. This computational assessment evaluates pharmacokinetic and safety profiles essential for therapeutic development, particularly addressing the translational potential from promising binding affinities to clinical applications (Table 12 and Fig. 9).

**Table 12:** Predicted ADMET, physicochemical, and toxicological profiles of selected flavonoids

Name	Agnuside	Casticin	Luteolin-6-C-glucoside	Orientin	Vitexin
Smiles	O = C(OCC1 = C[C@@H](O)[C@@H]2C = CO[C@@H](O[C@@H]3O[C@H](CO)[C@@H](O)[C@H](O)[C@H]3O)[C@H]12)c1ccc(O)cc1	COc1ccc(-c2oc3cc(OC)c(OC)c(O)c3c(= O)c2OC)cc1O	O = c1cc(-c2ccc(O)c(O)c2)oc2cc(O)c([C@@H]3O[C@H](CO)[C@@H](O)[C@H](O)[C@H]3O)c(O)c12	O = c1cc(-c2ccc(O)c(O)c2)oc2c([C@@H]3O[C@H](CO)[C@@H](O)[C@H](O)[C@H]3O)c(O)cc(O)c12	O = c1cc(-c2ccc(O)cc2)oc2c([C@@H]3O[C@H](CO)[C@@H](O)[C@H](O)[C@H]3O)c(O)cc(O)c12
LogS	− 2.176	− 4.077	− 3.477	− 3.697	− 3.78
LogD	0.277	2.525	− 0.147	− 0.024	0.145
LogP	0.511	2.772	0.708	0.757	1.232
Pgp-inh	0.001	0.998	0.009	0.007	0.005
Pgp-sub	0.348	0.001	0.993	0.99	0.991
HIA	0.692	0.015	0.909	0.912	0.841
F(20%)	0.016	0.003	0.991	0.954	0.934
F(30%)	0.996	0.003	1	1	1
Caco-2	− 6.126	− 4.813	− 6.251	− 6.293	− 6.222
MDCK	5.54E − 05	2.49E − 05	4.52E − 06	5.09E − 06	5.47E − 06
BBB	0.241	0.006	0.011	0.013	0.017
PPB	38.61%	84.41%	90.58%	88.77%	89.60%
VDss	0.379	0.823	0.834	0.842	0.903
Fu	38.79%	21.81%	14.30%	14.28%	11.41%
CYP1A2-inh	0.039	0.583	0.156	0.199	0.211
CYP1A2-sub	0.025	0.979	0.041	0.039	0.039
CYP2C19-inh	0.018	0.313	0.021	0.02	0.023
CYP2C19-sub	0.067	0.381	0.05	0.048	0.053
CYP2C9-inh	0.007	0.727	0.049	0.071	0.067
CYP2C9-sub	0.098	0.824	0.264	0.241	0.526
CYP2D6-inh	0.006	0.106	0.03	0.028	0.094
CYP2D6-sub	0.103	0.809	0.162	0.158	0.167
CYP3A4-inh	0.064	0.487	0.043	0.058	0.065
CYP3A4-sub	0.074	0.427	0.011	0.011	0.02
CL	1.825	4.28	4.067	5.439	4.091
T12	0.932	0.808	0.823	0.833	0.765
hERG	0.023	0.089	0.214	0.126	0.11
H-HT	0.079	0.109	0.133	0.169	0.175
DILI	0.951	0.962	0.966	0.98	0.978
Ames	0.063	0.402	0.729	0.824	0.797
ROA	0.457	0.348	0.025	0.047	0.076
FDAMDD	0.038	0.054	0.019	0.01	0.008
SkinSen	0.085	0.261	0.92	0.903	0.67
Carcinogenicity	0.943	0.032	0.037	0.039	0.072
EC	0.003	0.003	0.003	0.003	0.003
EI	0.025	0.631	0.628	0.508	0.134
Respiratory	0.522	0.228	0.051	0.071	0.085
BCF	0.505	1.535	0.923	0.866	0.777
IGC50	3.401	3.768	4.172	4.097	4.139
LC50	4.568	5.215	4.991	5.136	5.152
LC50DM	5.043	6.465	5.747	5.326	5.23
NR-AR	0.119	0.071	0.039	0.063	0.115
NR-AR-LBD	0.696	0.115	0.299	0.288	0.288
NR-AhR	0.016	0.939	0.835	0.846	0.88
NR-Aromatase	0.02	0.879	0.889	0.909	0.932
NR-ER	0.735	0.204	0.605	0.569	0.603
NR-ER-LBD	0.282	0.585	0.693	0.827	0.812
NR-PPAR-gamma	0.004	0.93	0.794	0.556	0.465
SR-ARE	0.054	0.817	0.341	0.157	0.193
SR-ATAD5	0.013	0.874	0.387	0.185	0.259
SR-HSE	0.006	0.25	0.271	0.075	0.052
SR-MMP	0.276	0.897	0.868	0.802	0.798
SR-p53	0.397	0.954	0.883	0.708	0.782
MW	466.15	374.1	448.1	448.1	432.11
Vol	435.716	360.741	413.147	413.147	404.357
Dense	1.07	1.037	1.085	1.085	1.069
nHA	11	8	11	11	10
nHD	6	2	8	8	7
TPSA	175.37	107.59	201.28	201.28	181.05
nRot	7	5	3	3	3
nRing	4	3	4	4	4
MaxRing	9	10	10	10	10
nHet	11	8	11	11	10
fChar	0	0	0	0	0
0nRig	23	18	24	24	24
Flex	0.304	0.278	0.125	0.125	0.125
nStereo	9	0	5	5	5
NonBiodegradable	1	0	2	2	2
NonGenotoxic_Carcinogenicity	0	0	0	0	0
SureChEMBL	0	0	0	0	0
LD50_oral	0	0	0	0	0
Skin_Sensitization	2	5	7	7	3
Acute_Aquatic_Toxicity	2	0	3	3	3
Toxicophores	1	1	2	2	1
Genotoxic_Carcinogenicity_Mutagenicity	3	0	0	0	0
QED	0.219	0.702	0.247	0.247	0.3
Synth	4.644	2.465	4.06	4.072	3.954
Fsp3	0.5	0.211	0.286	0.286	0.286
MCE-18	88.636	20	91	91	87.63
Natural Product-likeness	2.234	1.265	2.027	2.014	1.978
Alarm_NMR	2	3	3	3	2
BMS	0	0	0	0	0
Chelating	0	1	1	1	0
PAINS	0	0	1	1	0
Lipinski	Rejected	Accepted	Rejected	Rejected	Accepted
Pfizer	Accepted	Accepted	Accepted	Accepted	Accepted
GSK	Rejected	Accepted	Rejected	Rejected	Rejected
GoldenTriangle	Accepted	Accepted	Accepted	Accepted	Accepted

#### Absorption Properties

##### Solubility and Permeability

The LogS values reveal significant differences in aqueous solubility among the compounds. Casticin demonstrates the poorest solubility (− 4.077), while Agnuside shows the best (− 2.176), followed by Orientin (− 3.697), Luteolin-6-C-glucoside (− 3.477), and Vitexin (− 3.78). This pattern correlates with the glycosidic nature of compounds, where sugar moieties enhance water solubility compared to the aglycone Casticin.

Human intestinal absorption (HIA) predictions vary dramatically across compounds. Casticin shows extremely poor absorption (1.5%), likely due to its high lipophilicity (LogP = 2.772) and poor solubility. Conversely, the glycosidic compounds demonstrate excellent absorption potential: Luteolin-6-C-glucoside (90.9%), Orientin (91.2%), Vitexin (84.1%), and Agnuside (69.2%). This suggests that glycosylation, while reducing lipophilicity, maintains favorable absorption characteristics through active transport mechanisms.

##### Caco-2 Permeability

All compounds exhibit poor Caco-2 permeability (≤ − 4.813), indicating limited passive transcellular transport. This finding is consistent with their hydrophilic nature and suggests that absorption may rely on carrier-mediated transport rather than passive diffusion. The poor permeability values align with the compounds’ polar surface areas (TPSA) ranging from 107.59 (Casticin) to 201.28 Ų (Luteolin-6-C-glucoside and Orientin), exceeding optimal ranges for passive permeability.

#### Distribution Properties

##### Blood–Brain Barrier Penetration

BBB penetration presents a critical limitation for neurological applications. All compounds show poor BBB permeability: Casticin (0.6%), Vitexin (1.7%), Orientin (1.3%), Luteolin-6-C-glucoside (1.1%), and Agnuside (24.1%). Agnuside demonstrates the best CNS penetration potential, though still suboptimal for direct neurological targeting. This limitation may necessitate alternative delivery strategies or prodrug approaches for neurological applications.

##### Protein Binding and Volume of Distribution

Protein binding varies significantly, with Luteolin-6-C-glucoside showing the highest binding (90.58%), followed by Vitexin (89.60%), Orientin (88.77%), Casticin (84.41%), and Agnuside (38.61%). High protein binding may limit free drug concentrations but could provide sustained exposure. Volume of distribution (VDss) values indicate moderate tissue distribution for most compounds, with Vitexin showing the highest (0.903 L/kg).

#### Metabolism and Excretion

##### CYP450 Interactions

The compounds demonstrate varied CYP450 interaction profiles. Casticin emerges as the most problematic, with high inhibition potential for CYP1A2 (58.3%), CYP2C9 (72.7%), CYP2D6 (10.6%), and CYP3A4 (48.7%), suggesting significant drug-drug interaction risks. The glycosidic compounds show more favorable profiles, with minimal inhibition across CYP enzymes, indicating reduced potential for metabolic drug interactions.

Substrate predictions suggest that most compounds may undergo metabolism primarily through CYP3A4 and CYP2C19 pathways, with glycosidic compounds showing lower substrate probabilities, potentially indicating slower metabolism and longer half-lives.

##### Clearance and Half-Life

Predicted clearance rates vary, with Orientin showing the highest (5.439 mL/min/kg) and Agnuside the lowest (1.825 mL/min/kg). Half-life predictions range from 0.765 h (Vitexin) to 0.932 h (Agnuside), suggesting relatively rapid elimination that may require frequent dosing or sustained-release formulations.

#### Toxicity Assessment

##### Cardiotoxicity

hERG inhibition, a predictor of cardiotoxicity, shows acceptable profiles for most compounds. Luteolin-6-C-glucoside presents the highest risk (21.4%), while other compounds show minimal inhibition (≤ 12.6%), indicating generally low cardiotoxic potential.

##### Hepatotoxicity

Drug-induced liver injury (DILI) predictions are generally favorable, with all compounds showing low hepatotoxicity risk (≤ 98%), except minor concerns with Orientin and Vitexin. This suggests acceptable hepatic safety profiles for therapeutic development.

##### Mutagenicity and Carcinogenicity

Ames test predictions indicate low mutagenic potential across all compounds (≤ 82.4%), with Luteolin-6-C-glucoside and Orientin showing the lowest risk. Carcinogenicity assessments suggest minimal concern, supporting their safety profiles for long-term therapeutic use.

#### Drug-Likeness Assessment

##### Rule of Five Compliance

The compounds show mixed compliance with Lipinski’s Rule of Five. Molecular weights range from 374.1 (Casticin) to 466.15 (Agnuside), with Agnuside exceeding the 500 Da threshold. TPSA values exceed 140 Ų for glycosidic compounds, potentially limiting oral bioavailability. However, natural product origins may provide alternative absorption pathways not captured by traditional rules.

##### Synthetic Accessibility and Drug-Likeness

QED (Quantitative Estimate of Drug-likeness) scores vary significantly: Casticin (0.702), Vitexin (0.300), Agnuside (0.219), Luteolin-6-C-glucoside, and Orientin (0.247). Casticin shows the most favorable drug-like properties, while glycosidic compounds require optimization for improved drug-likeness.

##### Structure–Activity Relationships

The ADMET analysis reveals clear structure–activity relationships. Glycosylation improves solubility and absorption but reduces BBB penetration and traditional drug-likeness metrics. Casticin’s aglycone structure provides better lipophilic balance but suffers from poor solubility and high CYP inhibition potential.

### Modulatory Effects of Vitex A-C, Prami, and Their Combination Against the Impaired Locomotor Activity, Muscular Coordination, and Anxiety Behavior Induced by ROT in Rats

**Fig. 10:**
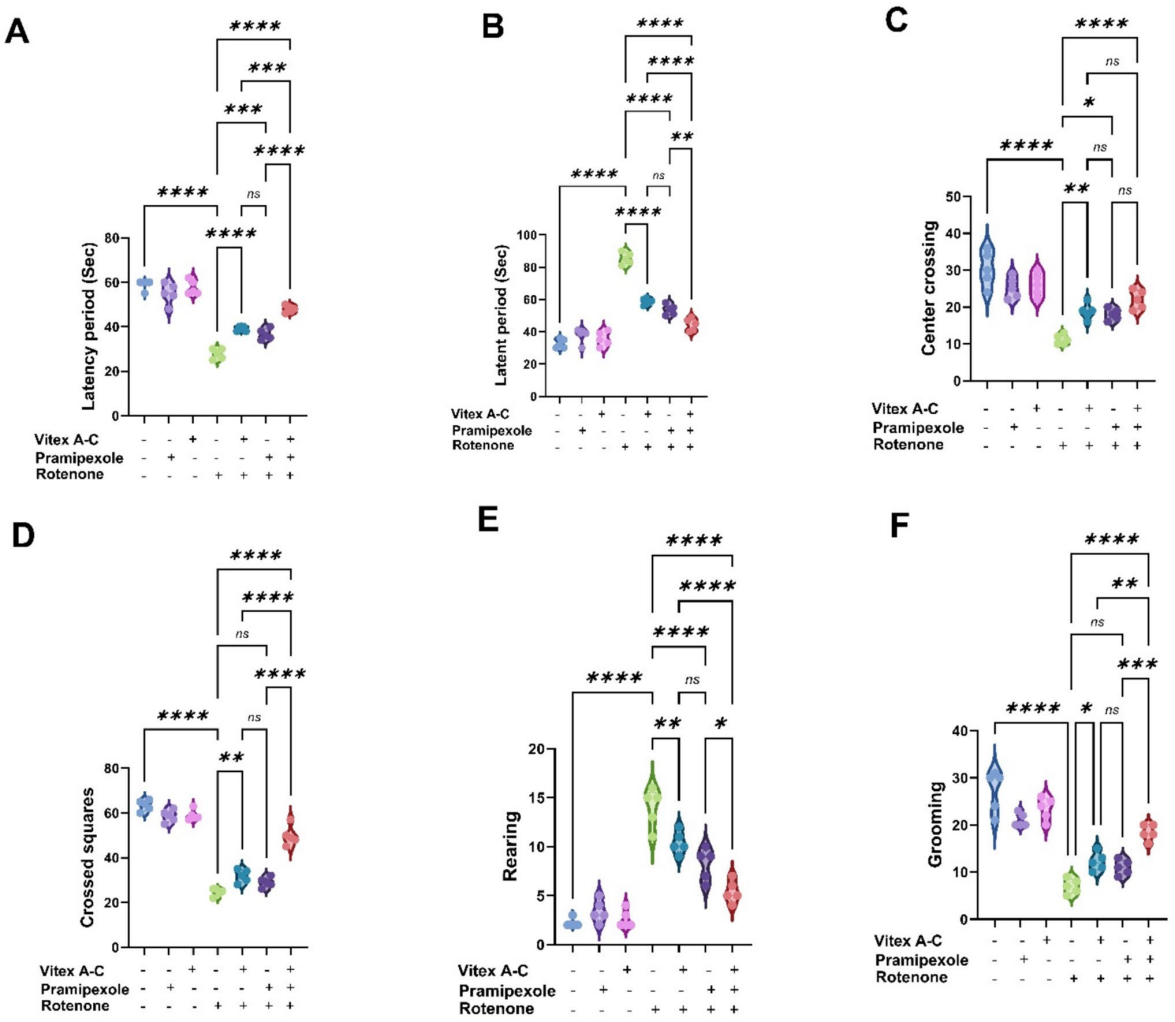
Effects of Vitex agnus-castus (Vitex A-C) and pramipexole (Prami) or their combination in the striatum of rotenone (ROT)-induced neurobehavioral alterations in male rats. **A** Rotarod latency period. **B** Open field latent period. **C** Open field center crossing. **D** Open field crossed square. **E** Open field rearing. **F** Open field grooming. Bars expressed mean ± SE (n = 5). Data analyzed via one-way ANOVA followed by Tukey’s post-hoc test. Significance represented as ns=non-signifiicant, * < 0.05%, ** < 0.01%, *** < 0.001%, and **** < 0.0001%

As illustrated in Fig. 10, in the neurobehavioral tests, the rotarod test’s latency time to fall did not change significantly among the control, Prami, and *Vitex A-C* groups. The latency period in the ROT-intoxicated group was noticeably downregulated by 52.88% in comparison with the control data. In contrast, the treated groups with *Vitex A-C* extract, Prami, and combination cotreated groups showed significant elevation in the latency period by 40.29%, 31.65%, and 72.66%, respectively, relative to the ROT-intoxicated group.

According to the open field parameters, latent period, central crossing, crossed squares, rearing, and grooming, no notable variations were observed in the aforementioned parameters among Control, Prami, and *Vitex A-C* groups. The ROT group exhibited an observable increase in the latent period and rearing by 161.96% and 536.36%, respectively, as compared to the control level. Conversely, there were noticeable downregulations in center crossing, crossed square, and grooming data by 63.16%, 61.20%, and 74.26% relative to the control group. The *Vitex A-C*, Prami, and combination co-treated groups revealed a noticeable decrease in the latent period (by 32.1%, 37.24%, and 48.01%) and rearing (by 25.71%, 41.43%, and 61.43%), respectively, when compared to the ROT-intoxicated group. Center square (by 67.86%, 60.71%, and 96.43%), crossed squares (by 29.27%, 17.89%, and 99.18%), and grooming (by 74.29%, 57.14%, and 162.86%) were substantially elevated in *Vitex A-C*, Prami, and combination co-treated groups in comparison with the ROT-intoxicated group, as revealed in Fig. 10.

### Modulatory Effects of Vitex A-C, Prami, and Their Combination on Dopamine Changes Induced by ROT in the Rat’s Striatum

**Fig. 11:**
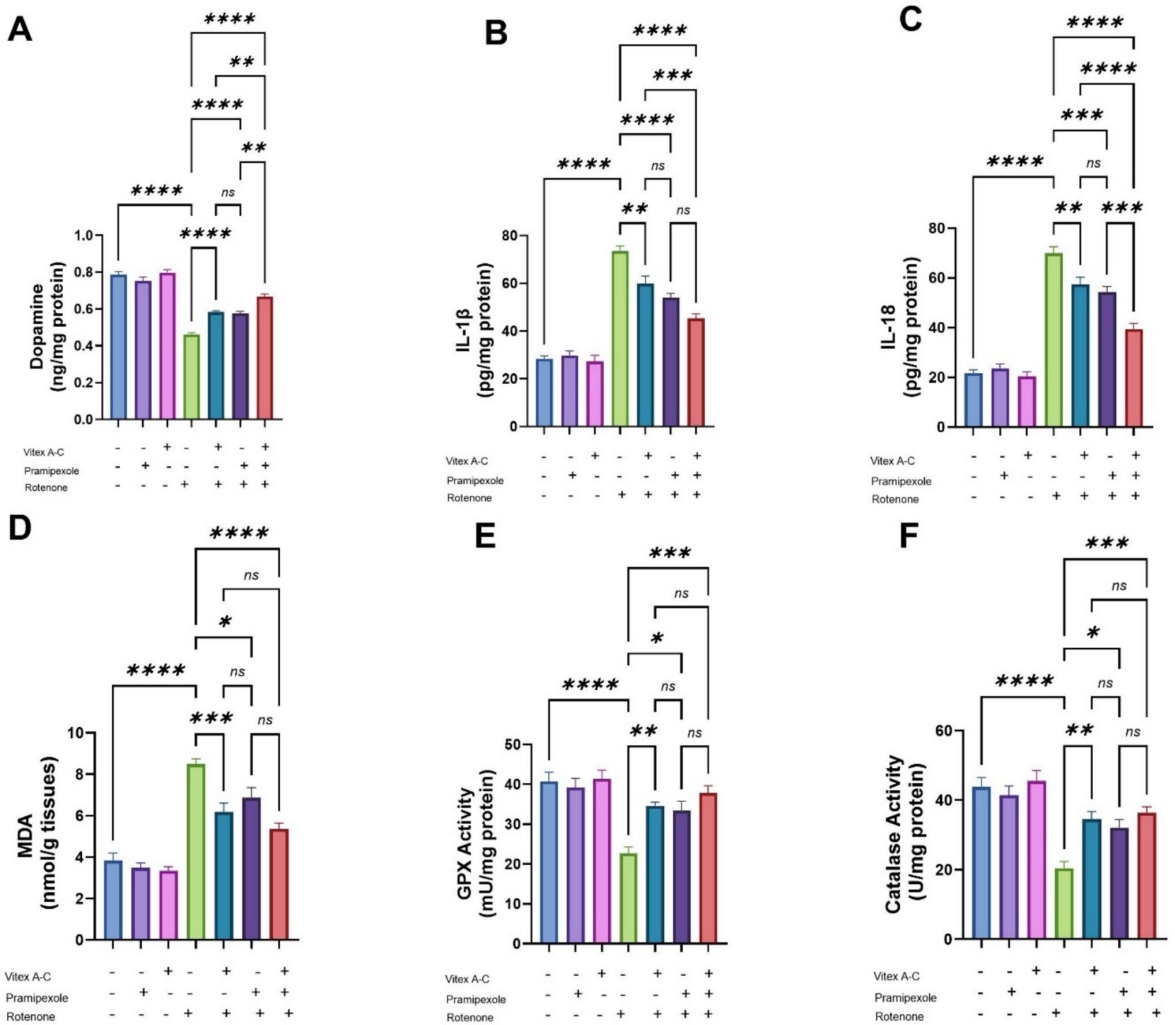
The neurobiochemical protective effects of Vitex agnus-castus (Vitex A-C) and pramipexole (Prami) or their combination on neurotoxicity induced by rotenone (ROT) in rats’ striatum. **A** Dopamine, **B** IL-1β (interleukin-1β), **C** IL-18 (interleukin-18), **D** MDA (malondialdehyde) levels, **E** GPX (glutathione peroxidase), and **F** catalase activities. Bars expressed mean ± SE (n = 5). Data analyzed via one-way ANOVA followed by Tukey’s posthoc test. Significance represented as ns=non-signifiicant, * < 0.05%, ** < 0.01%, *** < 0.001%, and **** < 0.0001%

According to dopamine concentration, there was a noticeable decline in the dopamine concentration (41.4%) in the striatal tissue of ROT-intoxicated rats relative to controls. In comparison to the ROT-group, the co-treated ones with *Vitex A-C*, Prami, and their combination revealed a detectable increase in the dopamine level by 26.4%, 24.7%, and 44.6%, respectively. The greatest increment was observed in the combined group, then the *Vitex A-C* extract and the Prami co-treated groups. However, no noticeable variations were seen in dopamine levels between the control group, the Prami, and the *Vitex A-C* medicated groups (Fig. 11 A).

### Modulatory Effects of Vitex A-C, Prami, and Their Combination Against Changes in Proinflammatory Cytokines Induced by ROT in the Rat’s Striatum

Concerning proinflammatory cytokines, IL-1β and IL-18 striatal levels substantially increased by 159% and 221.7%, respectively, in the ROT-treated group, contrasting with the control, while exhibiting a significant decrease in co-treated groups with *Vitex A-C* extract (18.6% and 18.1%, respectively), Prami (26.4% and 22.3%, respectively), and their combination (38.4% and 43.8%, respectively), if compared with the untreated one. The greatest decrements were noticed in the combined group, followed by Prami and *Vitex A-C* cotreated ones. Conversely, no discernible variations were seen in those cytokines between the control group, the Prami, and the *Vitex A-C* treated groups (Figs. 11B and  11C).

### Modulatory Effects of Vitex A-C, Prami, and Their Combination Against Redox Imbalance Induced by ROT in the Rat’s Striatum

The striatal tissue of ROT-treated rats displayed a significant increase in MDA (121%) levels with a substantial decrease in GPX (44.2%) and CAT (53.2%) activities, contrasting with the control rats, while the cotreatment with *Vitex A-C*, Prami, and their combination significantly lessened MDA levels (27.3%, 12.2%, and 36.8%, respectively), relative to untreated rats. Also, they significantly increased GPX activity (46%, 20.7%, and 66.6%, respectively) and CAT activity (49.4%, 17.7%, and 78%, respectively) if compared to the ROT-treated group. The most oxidant status improvements were observed in the combination group, followed by *Vitex A-C*, and then the Prami co-treated groups. However, when comparing the Prami, *Vitex A-C*, and control groups, no discernible changes were seen in the previously indicated metrics (Fig. 11D–F).

### Modulatory Effects of Vitex A-C, Prami, and Their Combination Against ROT-Induced Neuronal Pyroptosis in Rat Striatum

**Fig. 12:**
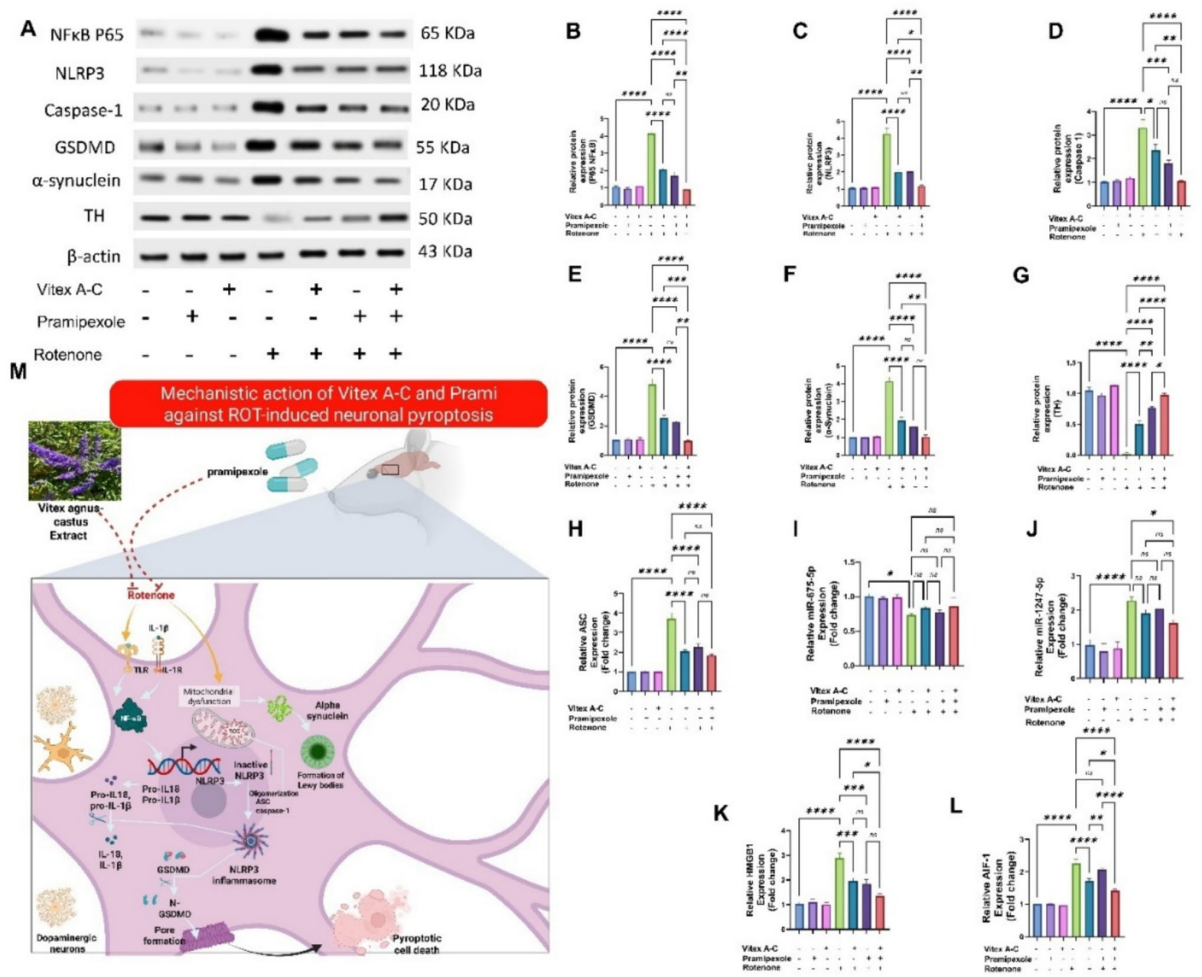
Effects of Vitex agnus-castus (Vitex A-C) and pramipexole (Prami) or their combination on neuronal pyroptosis induced by rotenone (ROT) in rats’ striatum. **A** Western blot bands representing the protein expression of nuclear factor kappa B (NFκB p65), NOD-like receptor protein 3 (NLRP3), GSDMD, caspase-1, α-synuclein, and tyrosine hydroxylase (TH) relative to β-actin. **B** Relative protein expression of NFκB p65. **C** Relative protein expression of NLRP3. **D** Relative protein expression of caspase-1. **E** Relative protein expression of GSDMD. **F** Relative protein expression of α-synuclein. **G** Relative protein expression of TH. **H** Relative mRNA expression of apoptosis-associated speck-like protein containing a CARD(ASC). **I** Relative mRNA expression of miR-675-5p. **J** Relative mRNA expression of miR-1247-5p. **K** Relative mRNA expression of high mobility group box 1 (HMGB1). **L** Relative mRNA expression of allograft inflammatory factor 1 (AIF-1). **M** Mechanistic action of Vitex A-C and Prami against ROT-induced neuronal pyroptosis. Bars expressed mean ± SE (n = 3). Data analyzed via one-way ANOVA followed by Tukey’s posthoc test. Significance represented as ns=non-signifiicant,* < 0.05%, ** < 0.01%, *** < 0.001%, and **** < 0.0001%

Figure 12 A illustrates the protein expression of NF-kB P65, NLRP3, GSDMD, caspase-1, α-synuclein, and TH from different experimental groups. As shown in Fig. 12B–G, *Vitex A-C* and Prami did not deviate much in the protein expression of NF-kB P65, NLRP3, GSDMD, caspase-1, α-synuclein, and TH from those in the control group. However, ROT group elicited remarkable increment in striatal protein expressions of NF-kB P65, NLRP3, GSDMD, caspase-1, and α-synuclein (by 290%, 304%, 371%, 224 and 315%, respectively) (Fig. 12B–F), along with a noticeable decline in striatal TH level (97.62%) (Fig. 12G), relative to control animals. Conversely, *Vitex A-C* and Prami or their combination exerted a noticible downregulation in the striatal levels of NF-kB P65 (50.31%, 58.97% and 78.05, respectively), NLRP3 (52.86%, 52.11% and 58.43%, respectively), GSDMD (47.79%, 53.18% and 79.70%, respectively), caspase-1 (28.97%, 45.42% and 68.24%, respectively), and α-synuclein (53.25%, 61.19% and 75.26%, respectively) (Fig. 12B–F), coupled with a noticible elevation in striatal TH levels (by 1.912-fold, 2.983-folds and 3.818-folds, respectively) (Fig. 12G), compared to ROT-intoxicated rats. Notably, the combined therapy has a significantly higher impact back to its normal values.

As illustrated in Fig. 12H, *Vitex A-C* and Prami did not significantly differ in ASC mRNA expression from those in the control group. However, ROT exerted an obvious upregulation in ASC mRNA expression by 274% contrasting to controls (Fig. 12H). In contrast, *Vitex A-C* and Prami or their combination produced a pronounced decline in ASC mRNA expression by 44.92%, 39.01%, and 50.56% (Fig. 12H) compared to the ROT group.

### Modulatory Effects of Vitex A-C, Prami, and Their Combination Against ROT-Induced miR-675-5p and miR-1247-5p Expression Changes in Rat’s Striatum

As illustrated in Fig. 12I and J, *Vitex A-C* and Prami were not substantially different in miR-675-5p and miR-1247-5p from those in the control group. ROT produced an obvious decline in striatal levels of miR-675-5p by 27.23% (Fig. 12I), along with a noteworthy upregulation in striatal levels of miR-1247-5p (by 134.7%) (Fig. 12 J), relative to the control group. On the other hand, *Vitex A-C* and Prami or their combination resulted in a nonsignificant change in the striatal levels of miR-675-5p (Fig. 12I), compared to the ROT-intoxicated group. *Vitex A-C* and Prami showed a non-significant variation in striatal miR-1247-5p levels, while combined therapy exhibited a noticeable downregulation in striatal miR-1247-5p levels (by 29%) (Fig. 12 J), compared to the ROT-intoxicated group.

### Modulatory Effects of Vitex A-C, Prami, and Their Combination Against ROT-Induced HMGB1 and AIF-1 Gene Expression Changes in Rat’s Striatum

As illustrated in Fig. 12 K and L, *Vitex A-C* and Prami did not significantly differ in HMGB1 and AIF-1 mRNA transcripts from those in the control group. However, ROT produced noteworthy upregulation in striatal levels of pro-inflammatory HMGB1 by 182.4% (Fig. 12 K) and AIF-1 (by 123.1%) (Fig. 12L) relative to the control group. On the contrary, *Vitex A-C* and Prami or their combination resulted in a prominent reduction in the striatal levels of HMGB1 (by 32.29%, 36.30%, and 53.18%, respectively) (Fig. 12 K) compared to the ROT-intoxicated group, while *Vitex A-C* and combination groups showed a noticeable downregulation in striatal AIF-1 levels (by 24.04% and 37%, respectively) (Fig. 12L), without any significant change in Prami group compared to the ROT-intoxicated group.

### Modulatory Effects of Vitex A-C, Prami, and Their Combination Against ROT-Induced Histopathological Alterations in Rat’s Striatum

**Fig. 13:**
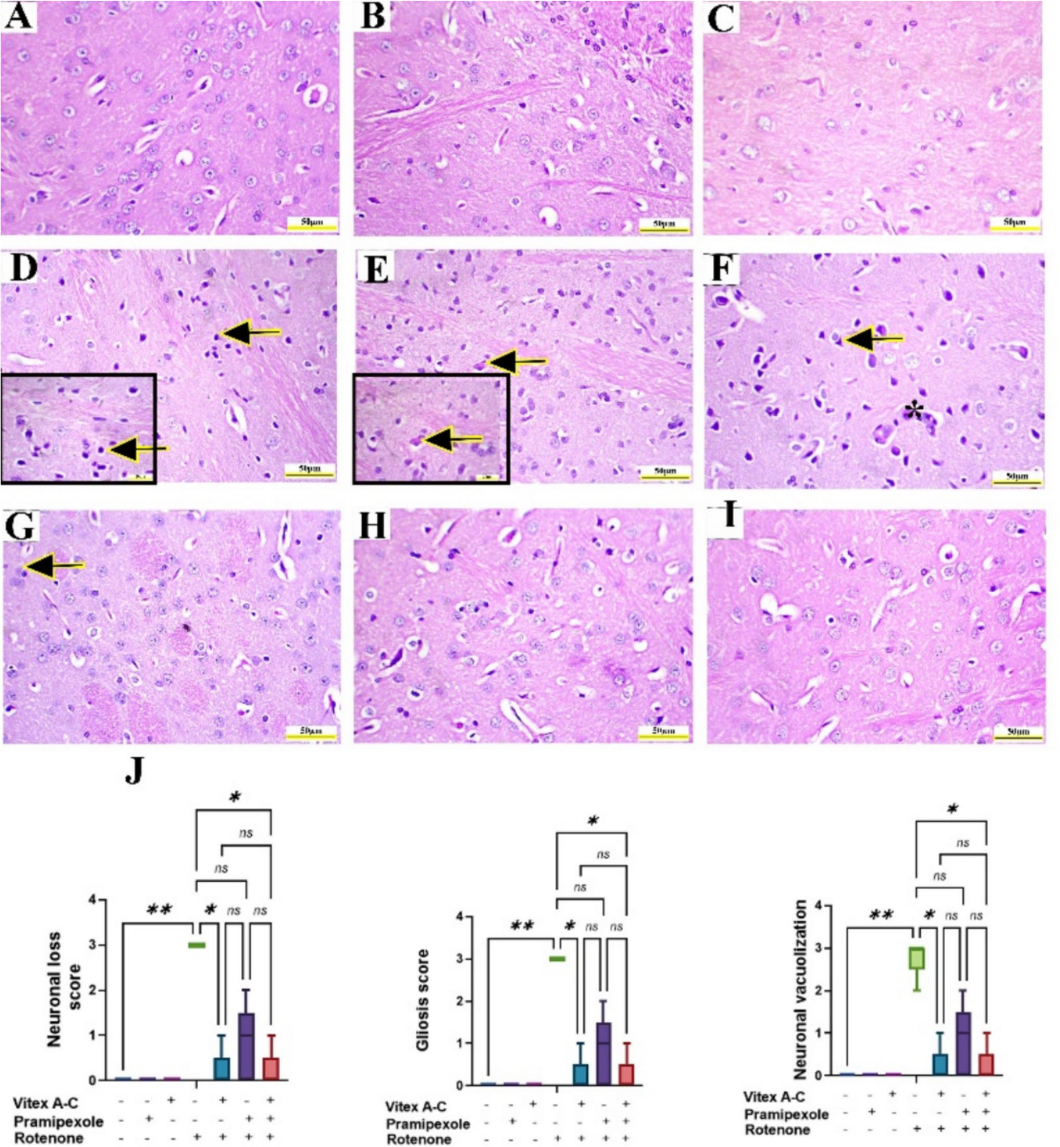
Effects of Vitex agnus-castus (Vitex A-C) and pramipexole (Prami) or their combination on histopathological changes induced by rotenone (ROT) in rats’ striatum. **A** Control group, **B** Prami control group, and **C** Vitex A-C control group showing normal structure of striatal neurons without any glial reaction. **D**–**F** ROT group showing **D** shrunken pyknotic neurons (arrows, inset), **E** Lewy-like body (arrow, inset) , and **F** Glial reaction including neuronophagia (arrow) and perivascular lymphocytic cuffing (*). **G** Vitex A-C + ROT-treated group showing improvement of striatal neuronal structure with mild neuronal shrinkage and glial reaction (arrow). **H** Prami + ROT-treated group showing restoration of striatal structure with moderate pyknosis in striatal neurons (arrow). **I** A combination group showing complete restoration of striatal histoarchitecture. **J** Neuronal loss, gliosis, and vacuolization histopathologic scores. Hematoxylin and eosin (H&E). Scale bar = 50 μm and inset D&E = 20 μm. Bars expressed mean ± SE (n = 5). Data was analyzed using the Kruskal–Wallis test followed by Dunn’s posthoc test. Significance represented as ns=non-significant, * < 0.05%, ** < 0.01%,

Microscopic examination of control, control Prami, and control *Vitex A-C* showed typical striatal neuronal histoarchitecture with vesicular nuclei without glial reaction (Fig. 13A–C). Conversely, the ROT-treated group showed extensive neuronal degeneration with Lewy-like body formation in addition to neuronal necrosis with satellitosis and neuronophagia as well as perivascular lymphocytic cuffing (Fig. 13D–F), whereas the *Vitex A-C* cotreated groups showed restoration of neuronal structure with mild neuronal pyknosis and glial reaction, while and Prami cotreated groups showed mild to moderate neuronal shrinkage (Fig. 13G and H). Furthermore, the combination group exhibited nearly normal striatal histoarchitecture (Fig. 13I). Neuronal degeneration statistical score showed a significant increment in ROT-treated animals relative to control rats (Fig. 13 J), whereas *Vitex A-C* and Prami did not show any significant change in all measured scores relative to the ROT group, whereas combined therapy showed a marked reduction in all measured scores relative to ROT-treated animals (Fig. 13 J).

### Modulatory Effects of Vitex A-C, Prami, and Their Combination Against ROT-Induced Immunohistochemical Changes (Caspase-1, GFAP, and Synaptophysin) in Rat’s Striatum

**Fig. 14:**
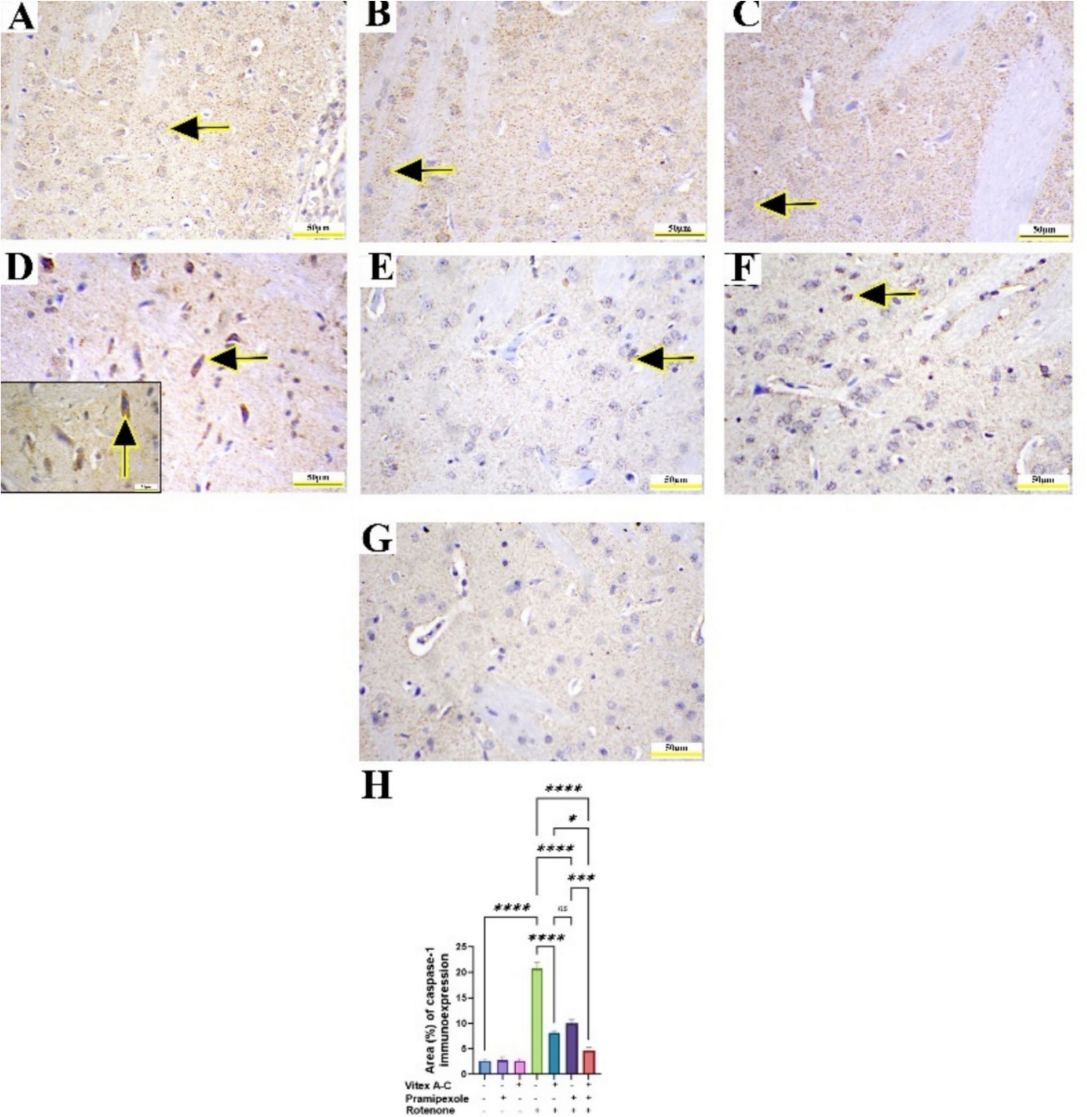
Effects of Vitex agnus-castus (Vitex A-C) and pramipexole (Prami) or their combination on caspase-1 immunoreactivity against rotenone (ROT) induced neurotoxicity in rats’ striatum. **A** Control group showing negative cytoplasmic caspase-1 immunoreactivity in neurons (arrow). **B** Prami control group showing negative cytoplasmic caspase-1 immunoreactivity in neurons(arrow). **C** Vitex A-C control group showing negative cytoplasmic caspase-1 immunoreactivity in neurons(arrow). **D** ROT group showing brown cytoplasmic staining denotes caspase-1 positivity (arrows, inset) in striatal neurons. **E** Vitex AC + ROT-treated group showing minimal caspase-1 immunoreactivity (arrow). **F** Prami + ROT-treated group showing mild caspase-1 immunoreactivity (arrow). **G** Combination group showing nearly negative caspase-1 immunoreactivity. **H** Area% % of caspase-1 immunoreactivity. Scale bar = 50 μm and inset D = 20 μm. Bars expressed mean ± SE (n = 5). Data analyzed via one-way ANOVA followed by Tukey’s post-hoc test. Significance represented as ns=non-significant, * < 0.05%, *** < 0.001%, and **** < 0.0001%

Immunohistochemical staining of caspase-1 in control, control Prami, and control *Vitex A-C* showed negative immunoreactivity in striatal neurons (Fig. 14A–C). Conversely, the ROT-treated group showed an intense caspase-1 immunoexpression in striatal neurons (Fig. 14D), whereas the *Vitex A-C* cotreated group showed a minimal caspase-1 immunoexpression, and Prami cotreated rats showed a mild caspase-1 immunoexpression (Fig. 14E and F). Furthermore, the combination group exhibited a negative caspase-1 immunoexpression (Fig. 14G). Semiquantitative analysis of caspase-1 area % showed a marked increase in its reactivity in ROT-treated animals relative to controls (Fig. 14H), whereas *Vitex A-C*, Prami, and combined therapy showed a marked reduction in caspase-1 immunoexpression relative to ROT-treated animals with more reduction in combination therapy (Fig. 14H).

**Fig. 15:**
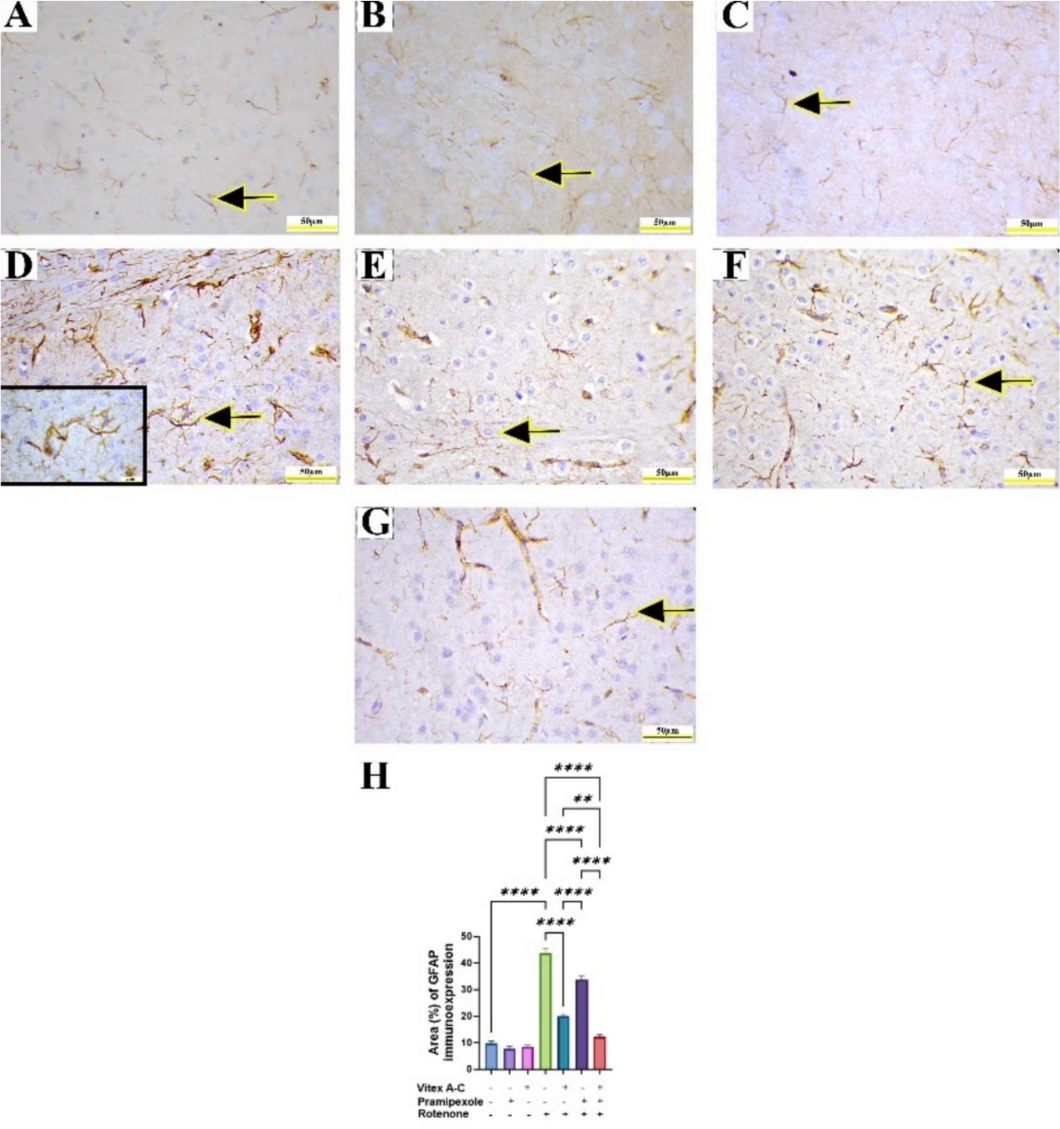
Effects of Vitex agnus-castus (Vitex A-C) and pramipexole (Prami) or their combination on glial fibrillary acidic protein (GFAP) immunoexpression in rotenone (ROT) induced neurotoxicity in rats’ striatum. **A** Control group showing minimal GFAP immunoreactivity (arrow). **B** Prami control group showing minimal GFAP immunoreactivity (arrow). **C** Vitex A-C control group showing minimal GFAP immunoreactivity (arrow). **D** ROT group showing an intense brown staining of GFAP denotes reactive astrocytes (arrow, inset) in striatal astrocytes. **E** Vitex A-C + ROT-treated group showing mild GFAP immunoreactivity (arrow). **F** Prami + ROT-treated group showing moderate GFAP immunoreactivity (arrow). **G** Combination group showing nearly minimal GFAP immunoreactivity (arrow). **H** Area% % of GFAP immunoreactivity. Scale bar = 50 μm and inset D = 20 μm. Bars expressed mean ± SE (n = 5). Data analyzed via one-way ANOVA followed by Tukey’s post-hoc test. Significance represented as ns=non-significant, ** < 0.01%, and **** < 0.0001%

Immunohistochemical staining of GFAP in control, control Prami, and control *Vitex A-C* showed minimal brown positive immunoreactivity of GFAP in astrocytic processes (Fig. 15A–C). Conversely, the ROT-treated group showed an intense GFAP immunoexpression in reactive striatal astrocytes (Fig. 15D), whereas the *Vitex A-C* cotreated group showed a mild GFAP immunoexpression, and Prami cotreated rats showed a moderate GFAP immunoexpression (Fig. 15E and F). Furthermore, the combination group exhibited a minimal GFAP immunoexpression (Fig. 15G). Semiquantitative analysis of GFAP area % showed a marked increase in its reactivity in ROT-treated animals relative to controls (Fig. 15H), whereas *Vitex A-C*, Prami, and combined therapy showed a marked reduction in GFAP immunoexpression relative to ROT-treated animals with more reduction in combination group (Fig. 15H).

**Fig. 16:**
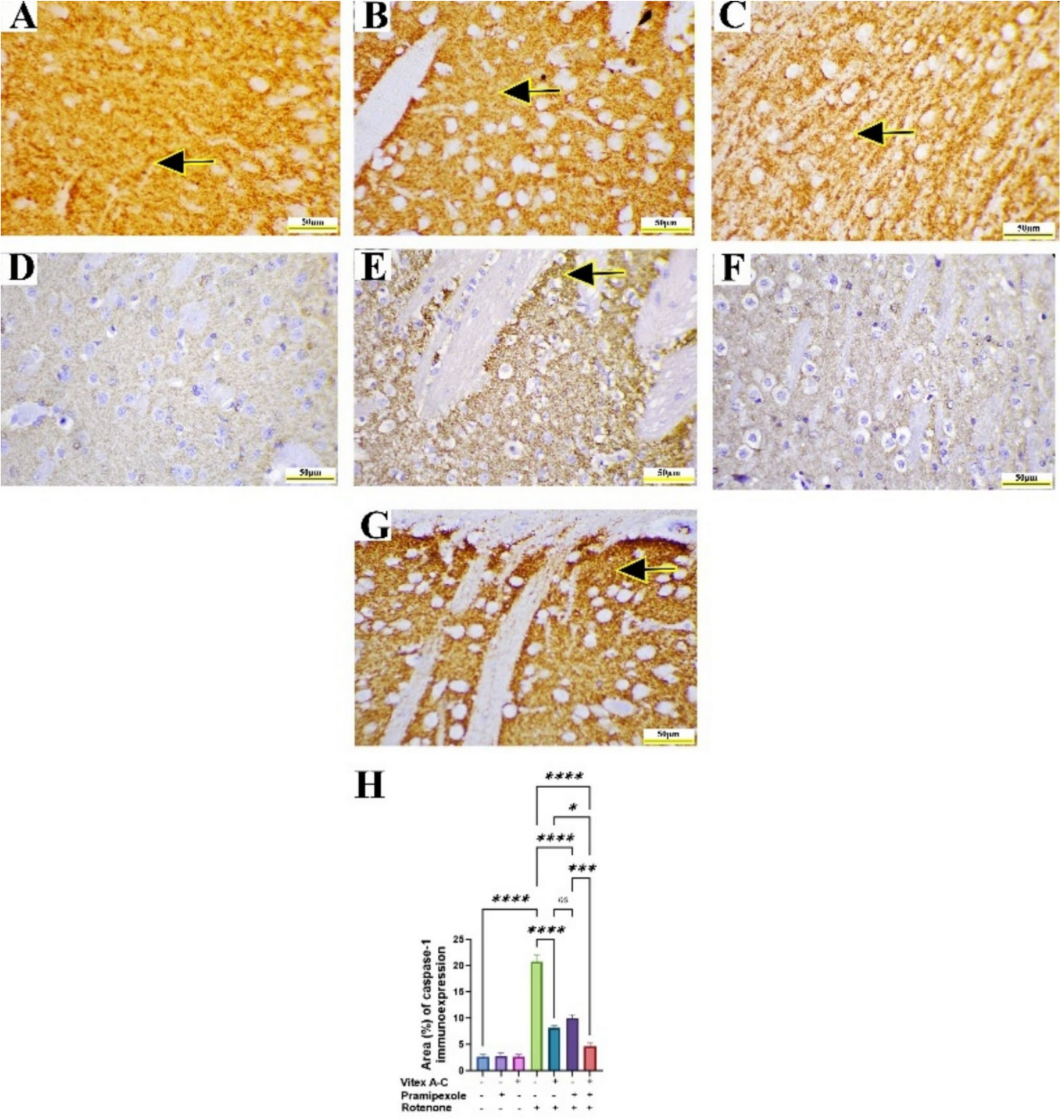
Effects of Vitex agnus-castus (Vitex A-C) and pramipexole (Prami) or their combination on synaptophysin immunoexpression in rotenone (ROT) induced neurotoxicity in rats’ striatum. **A** Control group showing a marked synaptophysin immunoreactivity (arrow). **B** Prami control group showing a marked synaptophysin immunoreactivity (arrow). **C** Vitex A-C control group showing a marked synaptophysin immunoreactivity (arrow). **D** ROT group showing a negative synaptophysin immunoexpression in the striatum. **E** Vitex A-C + ROTtreated group showing a moderate synaptophysin immunoreactivity (arrow). **F** Prami + ROTtreated group showing a mild synaptophysin immunoexpression. **G** Combination group showing an intense synaptophysin immunoexpression (arrow). **H** Area% % of synaptophysin immunoreactivity. Scale bar = 50 μm . Bars expressed mean ± SE (n = 5). Data analyzed via one-way ANOVA followed by Tukey’s post-hoc test. Significance represented as ns=non-significant, * < 0.05%, *** < 0.001%, and **** < 0.0001%

Immunohistochemical staining of synaptophysin in control, control Prami, and control *Vitex A-C* showed a marked brown positive immunoreactivity (Fig. 16A–C). Conversely, the ROT-treated group showed nearly negative synaptophysin immunoreactivity in striatal tissue (Fig. 16D), whereas the *Vitex A-C* cotreated group showed a moderate synaptophysin immunoreactivity in striatal tissue, and Prami cotreated rats showed a mild synaptophysin immunoreactivity in striatal tissue (Fig. 16E and F). Furthermore, the combination group exhibited an intense positive brown synaptophysin immunoreactivity in striatal tissue (Fig. 16G). Semiquantitative analysis of synaptophysin area % showed a substantial decline in its reactivity in ROT-treated animals relative to controls (Fig. 16H), whereas *Vitex A-C*, Prami, and combined therapy showed a marked upregulation in synaptophysin immunoexpression relative to ROT-treated animals with a higher increment in combination therapy (Fig. 16H).

## Discussion

Previous reports indicated that ROT can slowly simulate and progress various pathological changes in the brain tissue of some neuronal diseases [60, 61]**.** In this study, we found that intraperitoneal injection of male rats with ROT induced a variety of neurobehavioral, biochemical, and pathological changes in brain tissue. The mechanism by which many of these alterations arise is the creation of oxidative stress by ROT. And nowadays, phytochemicals are an excellent alternative for chemicals and drugs, which are often associated with negative side effects [62, 63]**.** So, we studied the possible improvements related to *Vitex A-C* extract, Prami, and their combination against ROT-induced neurotoxicity.

Current investigation exhibited significant deterioration in the ROT group in open field locomotor activity parameters involving the latent period, crossed squares, rearing, and the anxiety parameters (center crossing and grooming). Also, this study showed significant impairment of the muscular coordination, that confirmed by a significant downregulation in the mean of latency period in the rotarod test in comparison with the control group. Our results were in harmony with Manimarana et al. [64], who found that ROT treatment was correlated with weakness in the rotarod test’s muscle coordination and the open field test’s locomotor activity. Furthermore, Eddin et al. [65] noted that rats given ROT spent less time on the rotarod. According to the positive impact of *Vitex A-C* extract, there were several studies that supported our results, as De Oliveira et al. [66] proved that administration of Vitexin in pentylenetetrazole kindled rodents did not impair locomotor activity after being subjected to the rotarod test. As well as the same team reported in the same study that systemic administration of Vitexin has long-term neuroprotective effects via decreasing the severity of seizures induced by pentylenetetrazole in the rat model. Furthermore, Kang et al. [44] discovered that Prami enhanced spontaneous activity and decreased anxiety and sadness. It was hypothesized that the process could be connected to D2/D3 receptor activation in rats suffering from cerebral ischemia–reperfusion damage. It has been shown that mice’s voluntary behaviors are hindered by D2 receptor inhibition, malfunction, and the antidepressant. The neuroprotective effects of Prami may be related to the stimulation of D3 receptors [67] or may be via decreasing apoptosis through antioxidant and anti-inflammatory effects [68].

It was reported that neurotransmitter alterations can contribute to the pathophysiology of many neurodegenerative diseases [69]. The depletion of dopamine and selective deterioration of dopaminergic neurons are the most prevalent pathogenic characteristics of some neuronal diseases [69]. It was discovered that prolonged use of ROT inhibits dopamine D3 receptor expression and function [70, 71]. Our study proved that ROT injection significantly decreased dopamine levels in the striatal tissue of rats. These findings are in harmony with the study of Zhou et al. [72], who reported that chronic oral administration of ROT (30 mg/kg for 60 days) decreased striatal dopamine and its metabolites in C57BL/6 mice. Also, Khodir et al. [73] showed that male rats’ striatal dopamine levels significantly decreased after daily subcutaneous injection of ROT for 5 weeks. On the other hand, the co-treatment of ROT-treated rats with *Vitex A-C* extract significantly increased the dopamine levels. This could be because of the ability of *Vitex A-C* extract and its bioactive components to bind with dopamine receptors and induce dopaminergic activation [74]. Similarly, previous studies reported that *Vitex A-C* extract has many components that can interact with dopaminergic neurotransmitters, inducing their release and stimulating anxiolytic effects [74, 75]. Also, the co-treatment with Prami lesser increased the dopamine level than *Vitex A-C* extract. This relates to the fact that Prami serves as an agonist for D2/D3 dopamine receptors, causing dopamine receptors to release dopamine and altering some dopamine-mediated processes [76–78].

As ROT can selectively inhibit the activity of mitochondrial complex I and electron transport chain-1, which leads to ATP reduction and leakage of electrons that increase ROS by binding with O2 radicals, leading to the degenerative changes in the dopaminergic neurons [79, 80]. The buildup of ROS disrupts the oxidant balance, promotes oxidative stress, which raises MDA levels and suppresses the activities of antioxidant enzymes [72]. This is consistent with the present study as ROT treatment dramatically raised the striatal MDA while lowering GPX and CAT activities. Also, Manimarana et al. [64], Tseng et al. [81], and Khodir et al. [73] reported a noteworthy increase in MDA with decreased GPX and CAT activities in cortical and hippocampal, brain, and striatum tissue in albino Wistar rats intraperitoneally injected with ROT. However, the groups co-treated with both *Vitex A-C* and Prami improved the neurotoxic effects and recorded the highest antioxidant against ROT-induced neurotoxicity when compared with groups co-treated with *Vitex A-C* and Prami separately. These results appeared in our study with an observable decrease in striatal MDA and with a substantial increase in antioxidant enzymes. This may be due to the synergistic effects between the *Vitex A-C* extract and the Prami drug. According to the antioxidant effects of *Vitex* extract, there are previous studies that accord with us, as Ibrahim et al. [82] stated a significant decrease in lung MDA with an increase in antioxidant enzymes in lipopolysaccharide-intoxicated mice pretreated orally with *Vitex A-C* before intoxication. Also, El Kamari et al. [83] reported that *Vitex A-C* extract increased total antioxidant capacity and decreased the inflammation induced by carrageenan in rats. Deniz et al. [84] reported that pretreatment with *Vitex* decreased the total oxidant status with an increase in total antioxidant status against cisplatin-induced nephrotoxicity in male rats. Also, Ahangarpour et al. [85] showed that treatment with *Vitex* significantly decreased the uterine and ovarian MDA levels with increased SOD and CAT activities in aging mice. The antioxidant effects of Vitex may be attributed to its enrichment with components as flavonoids, triterpenoids, and glycosides that have antioxidant benefits, as reported by our analysis of *Vitex*. According to Prami effects, several studies have proved its protective effects. As Eisa et al. [86] reported that the treatment with Prami decreased sciatic nerve oxidative stress and inflammation in diabetic rats by decreasing MDA levels’ expressions with increasing GSH levels. Moreover, Salman et al. [87] found that the treatment with Prami decreased the brain MDA levels with increased GPX and SOD activities, besides GSH levels in rats exposed to traumatic brain injury. Also, Kang et al. [88] reported that Prami can reduce the hippocampal MDA and increase GSH levels in rats with brain ischemia/reperfusion damage. These antioxidant effects of Prami may be attributed to its capability to inhibit lipid peroxidation and free radical production [89].

The oxidative stress can induce the inflammatory process. Interestingly, caspase-1 cleavage is triggered when NF-κB activates the NLRP3 inflammasome, which cleaves the pyroptosis executive protein GSDMD’s C-terminal inhibitory domain, resulting in the creation of membrane holes that cause the cells to expand, lyse, and die [90–92] (Fig. 12 M). Activated NLRP3-dependent pyroptosis may further cause dopaminergic neuron damage, enhance inflammatory responses, and boost IL-1β production. This could be linked to ROT’s role in stimulating microglial activation and nuclear activation of NF-κB, which triggers the release of proinflammatory cytokines like IL-1β and others, resulting in neuroinflammation and degeneration, as documented by Le et al. [93]. Additionally, when ATP was present, ROT caused NLRP3 or ASC aggregates to form and NLRP3 to associate with ASC, which led to NLRP3-dependent caspase-1 stimulation [94]. Thus, rats given ROT consistently showed a clear rise in the protein expression of striatal GSDMD as well as NF-κB/NLRP3 level, caspase-1 activity, IL-1β, and IL-18 content. These results are consistent with Eddin et al. [65], who demonstrated that the intraperitoneal injection of ROT induced the expression of inflammatory mediators as IL-6, IL-1β, and TNF-α in rats. Also, Tseng et al. [81] reported a notable increment in brain IL-1β levels in rats subcutaneously injected with ROT. In addition, Khodir et al. [73] reported a considerable increase in striatal IL-1β and other proinflammatory cytokines in rats subcutaneously injected with ROT. On the other hand, the co-treatment with both *Vitex A-C* and Prami had anti-inflammatory and anti-apoptotic properties against ROT neurotoxicity relative to *Vitex A-C* and Prami separately. These results appeared in our study by a significant decrease in striatal GSDMD levels by inhibiting the NLRP3, caspase-1, IL-1β, and IL-18 signaling pathway. Concerning vitex, there are limited data illustrating the anti-pyroptotic neuroprotective activity of *Vitex A-C* extract, and this is considered the first report. The extract’s anti-inflammatory activity might be related to the plant’s antioxidant properties [83]. Also, it may be returned to the enrichment of *Vitex A-C* extract with components having antioxidant and anti-inflammatory characters, as Vitexin, Casticin, Orientin, Agnuside, and Luteolin-6-C-glucoside [95, 96], as proved in our findings after *Vitex* extract analysis. These components can reduce the deleterious effects of ROS and, consequently, inflammation by stopping free radical generation, accelerating their elimination, and boosting the antioxidant enzymes [97]. Several inflammatory pathways are counteracted by antioxidants because of their strong antioxidant capacity, as reported by Yahfoufi et al. [98]. The active ingredients of the plant can reduce inflammation by boosting anti-inflammatory factors, including Nrf2, and so reducing the production and release of certain pro-inflammatory chemokines [99]. Concerning to Prami, its anti-inflammatory and anti-pyroptotic activities may be attributed to its ability to regulate the Nrf2/HO-1 inflammatory pathway [32] and its antioxidant effects as previously mentioned. A study by Zhang et al. [100] showed that dexprami prevents lipopolysaccharide-induced neuroinflammation and cognitive impairments by protecting mitochondria and inhibiting the pyroptotic signal via inhibiting NLRP3 and caspase-1. Dong et al. [34] demonstrated the anti-inflammatory role of Prami that significantly decreased the levels of IL-1β in the mice’s substantia nigra in PD models. Additionally, Eisa et al. [86] reported that the treatment with Prami decreased sciatic nerve inflammation in diabetic rats by decreasing IL-β and TNF-α expressions. In fact, pyroptosis suppression was considered a useful mechanistic understanding and a possible treatment approach for neuroprotection against neurodegenerative diseases [101].

During the inflammatory process, HMGB1 is secreted from necrotic cells and binds to many receptors, which triggers the release of many cytokines that cause inflammation and the development of neurodegenerative diseases, including IL-6, TNFα, and IL-1β [102]. Also, it is believed that oxidative stress activates the HMGB1, which is substantially expressed in the PD model [103]. Rats injected with ROT upregulated proinflammatory HMGB1 and AIF-1. In consistent with our results, El-Sayed et al. [104] stated upregulation of proinflammatory HMGB1/TLR4 and Nrf2/HO-1 pathways in rats injected with ROT. Also, Dutta et al. [105] and Mohamed et al. [106] confirmed that exposure to ROT raised HMGB1 levels in preclinical PD models. Moreover, Chen et al. [107] explained that scorpion venom heat-resistant synthesized peptide significantly blocked the ROT-induced increase in levels of LPS and HMGB1 in the serum of ROT-treated mice. Furthermore, Elkady et al. [108] discovered that the intraperitoneal injection of ROT substantially raised the levels of HMGB1, TLR4, TNF-α, and NF-kB. HMGB1 regulates the expression of α-synuclein, while ROT exposure exerts a positively intensifying effect [109]. However, the levels of proinflammatory HMGB1 and AIF-1 were downregulated upon co-treatment with *Vitex A-C* extract and Prami. Regarding *Vitex A-C* extract, pretreatment with Vitexin inhibited HMGB1 release, preventing apoptosis and LPS-induced islet tissue damage in rats [110]. Also, Vitexin inhibited the gastric cancer in vivo and vitro by suppressing HMGB1-mediated activation of PI3K/Akt/HIF-1α signaling pathway [111]. In agreement with our results, Wang et al. [112] stated that the administration of Prami reduced the concentrations of TNF, IL-6, IL-1β, and HMGB1 in septic mice.

Numerous neurodegenerative illnesses, referred to as synucleopathies, have been linked to α-synuclein aggregation [113]. It has been shown that α-synuclein overexpression or aberrant aggregation may cause neuronal death, mitochondrial malfunction, and increased vulnerability to oxidative stress [114]. The pathophysiology of PD may be aided by the interaction of neuronal cells caused by abnormal α-synuclein aggregation [114]. In the present investigation, after administration of ROT, protein expression of α-synuclein was significantly higher, while ROT significantly reduced the TH level than the control value. In harmony with our investigation, those of Yuan et al. [115] revealed that ROT increased the intracellular aggregation and phosphorylation of α-synuclein in a calcium-dependent manner. Also, Silva et al. [116] reported that ROT-induced α-synuclein misfolding and aggregation. Moreover, in vivo, the chronic exposure of ROT caused the accumulation of α-synuclein and proteasomal impairment [117]. Furthermore, oral ROT administration caused surviving dopaminergic neurons to accumulate α-synuclein in their cytoplasm [118]. Also, ROT significantly reduced levels of TH protein in the olfactory bulb [119]. Aligning with our results, Luo et al. [120] stated that ROT decreased the number of TH-positive neurons in the SNc by approximately 60% in the control group. The neurotoxicity of ROT is marked by inhibition of TH in rat striatal slices, which is responsible for the dopamine deficiency [121]. Furthermore, Bai et al. [122] showed that ROT reduced TH expression in PC12 cells. Also, daily intraperitoneal injection of ROT caused α-synuclein accumulation and aggregation with loss of TH in the substantia nigra [6]. Additionally, ROT inhibited TH and increased α-synuclein expression in both SH-SY5Y cells and injected mice [123]. Similarly, the expression of α-synuclein in the *Vitex A-C* extract-treated rats was lower than that in the untreated group. Lima et al. [124] said that Vitexin, as a flavonoid, modulates gene transcription and phosphorylation of pathways of death and reduces neurodegeneration. To our knowledge, this is the first study showing the downregulating activity of *Vitex A-C* extract against α-synuclein aggregation, while, according to Prami, our study showed a significant decrease in α-synuclein with a significant increase in TH in the groups co-treated with Prami as compared to the untreated group. In agreement with these results, Luo et al. [125] showed that Prami reduces the quantity of α-synuclein accessible in the serum exosomes, helping to minimize a major pathogenic component in PD, in addition to relieving its symptoms. Also, Prami inhibits the phosphorylation of α-synuclein induced by inhibition of the ubiquitin proteasomal system [126]. Several neuroprotective properties of Prami were shown against the neurodegeneration brought on by ROT damage [118]. Moreover, in vivo, Prami attenuated the up-regulation of intracellular α-synuclein immunoreactivity in the substantia nigra of ROT-treated mice. Finally, in the spinal cord and striatum, Prami reversed the effects of autoimmune encephalomyelitis by restoring baseline levels of α-synuclein [127].

Numerous studies have shown that miRNAs affect the NLRP3-Nrf2 axis in PD. According to previous reports, miR-675-5p and miR-1247-5p are involved in several malignancies and neurological illnesses. In the present investigation, an obvious decline in striatal levels of miR-675-5p, besides an upregulation in miR-1247-5p levels, was reported in ROT-treated animals. Parallel to our report, Liang et al. [22] showed a considerable reduction in miR-675-5p expression in the striatum of PD animal models, but miR-1247-5p expression was greater. Also, they noted in PD neuron models, either down-regulated miR-1247-5p or up-regulated miR-675-5p controls inflammation and pyroptosis [22]. Furthermore, to the best of our knowledge, we are the first report showed that the combined therapy of both vitex and Prami exhibited a noticeable downregulation in striatal miR-1247-5p levels without any significant change in miR-675-5p levels relative to ROT-treated animals.

Although the molecular docking results provide strong computational evidence of potential interactions, the lack of experimental validation remains a limitation of this study. In particular, in vitro binding assays such as surface plasmon resonance (SPR), isothermal titration calorimetry (ITC), or microscale thermophoresis (MST) would be valuable to confirm the predicted binding affinities and kinetics. Future studies integrating these experimental approaches will be essential to substantiate and extend our computational findings.

## Conclusion

The molecular docking analysis performed in this study provides comprehensive insights into the potential therapeutic applications of bioactive compounds (Agnuside, Casticin, Luteolin-6-C-glucoside, Orientin, and Vitexin) against various targets implicated in neuroinflammatory and neurodegenerative processes. To establish clinical relevance, all findings are systematically compared with prami, a clinically established dopamine agonist used in PD treatment, which serves as our reference compound. Vitexin has a much higher binding affinity for alpha-synuclein than prami. Agnuside may also provide better dopaminergic regulation than the current clinical standard, which might lead to better treatment effectiveness in PD and other illnesses involving dopamine.

As underlying molecular pathomechanisms, this work revealed for the first time the critical involvement of dysregulation in the NLRP-3/caspase-1/GSDMD pyroptotic signal, miR-675-5p, and miR-1247-5p axis in ROT-evoked striatal neurodegenerative changes. Our results demonstrated that *Vitex A-C* and Prami reduced ROT-induced striatal neurodegeneration and its related dysregulation in oxidative stress, inflammatory, and pyroptotic signals, as well as its related histopathological changes. Also, the combined therapy showed a substantial decline in caspase-1 and GFAP immunoreactivity with an increment in synaptophysin immunoreactivity. The combined treatment’s positive effects were achieved via modulating the dopamine, oxidative markers, IL-18, IL-1β, HMGB1, AIF-1, NLRP-3/caspase-1/GSDMD, and miR-1247-5p without any substantial effect on miR-675-5p. It is interesting to note that the combination produced better results in all of the previously stated biochemical markers and histological findings than monotherapy. Taking into account our findings provided preclinical evidence regarding the usage of this combined regimen as a promising therapeutic approach for alleviation of ROT-triggered striatal neurodegeneration. 

## Supplementary Information

Below is the link to the electronic supplementary material.

ESM 1 (ZIP 222 KB)

## Data Availability

The data are available from the corresponding author with a reasonable request.
