# Supplementary figures and images for: Deciphering the Role of NLRP-3/Caspase-1/GSDMD Pyroptotic Signal, miR-675-5p, and miR-1247-5p in Mitigation of Neurobehavioral and Neuropathological Alterations in Rotenone-Induced Striatal Neurodegeneration by Vitex agnus-castus Leaf Extract and/or Pramipexole in Male Rats
Source: Mol Neurobiol. 2025 Dec 6;63(1):265. doi: 10.1007/s12035-025-05405-3 (PMC12681498; doi:10.1007/s12035-025-05405-3)

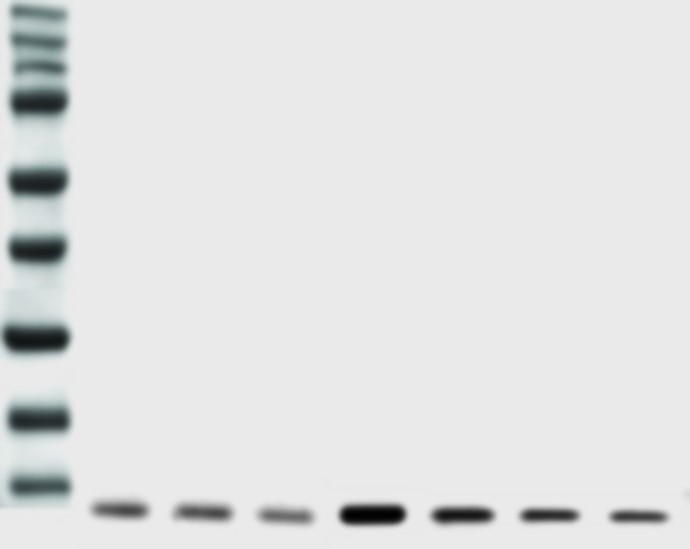

Supplement: Supplementary file 1 — (ZIP 222 KB) [file 12035_2025_5405_MOESM1_ESM.zip › western/alpha synuclein 1.jpg]

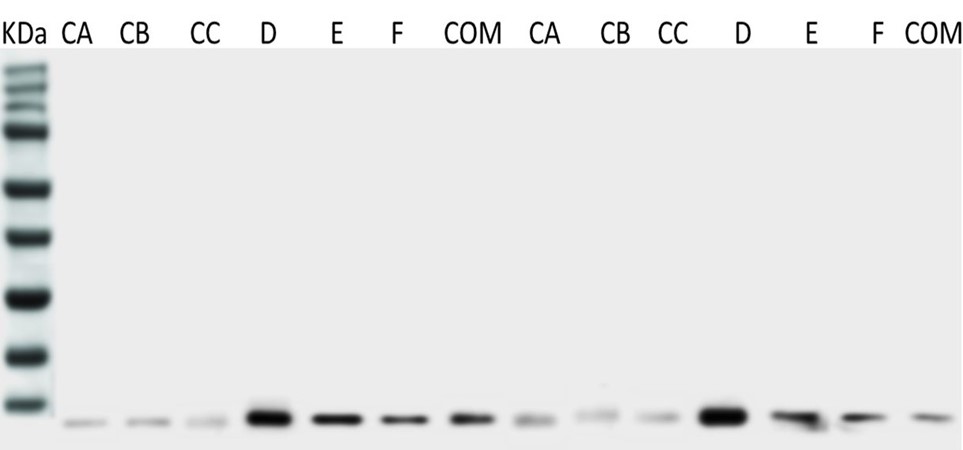

Supplement: Supplementary file 1 — (ZIP 222 KB) [file 12035_2025_5405_MOESM1_ESM.zip › western/alpha synuclein 2 and 3.jpg]

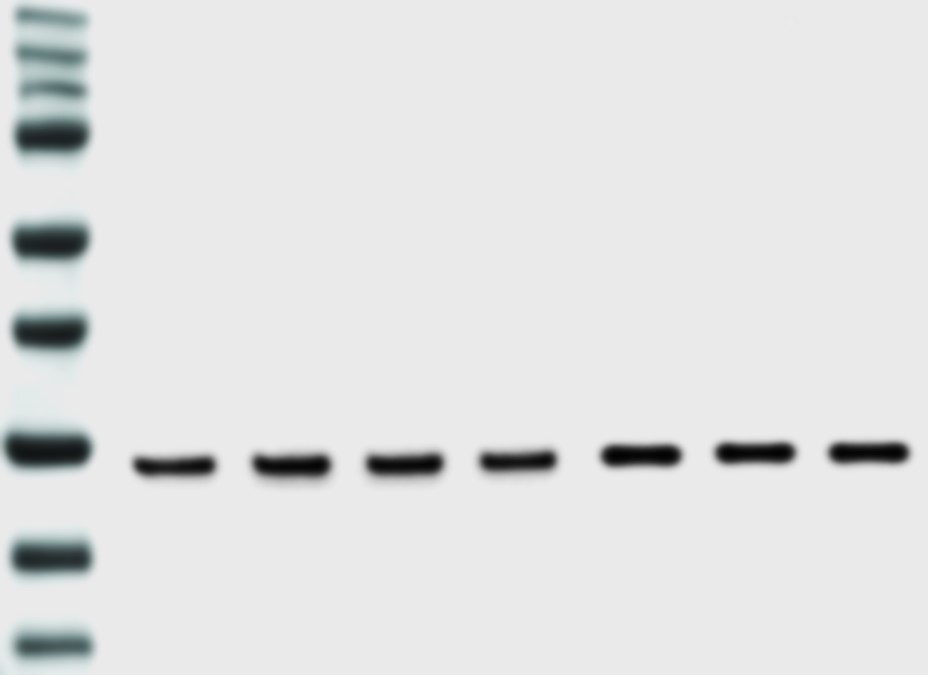

Supplement: Supplementary file 1 — (ZIP 222 KB) [file 12035_2025_5405_MOESM1_ESM.zip › western/BETA ACTIN 1.jpg]

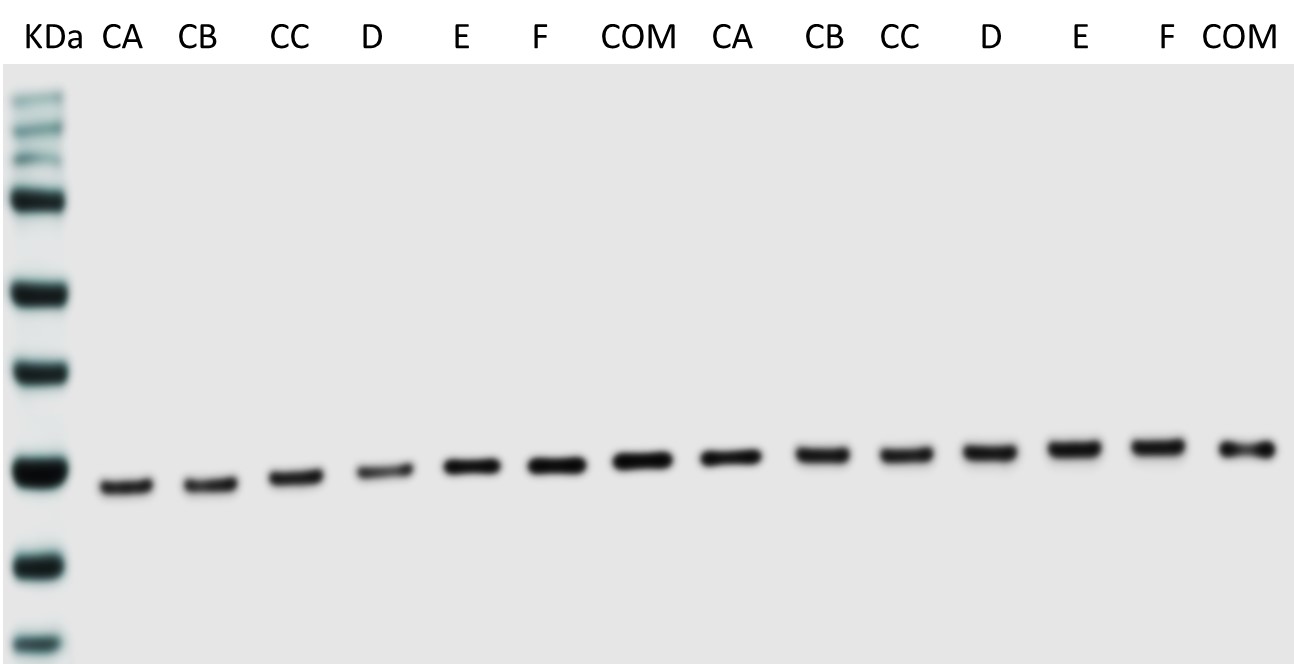

Supplement: Supplementary file 1 — (ZIP 222 KB) [file 12035_2025_5405_MOESM1_ESM.zip › western/BETA ACTIN 2 AND 3.jpg]

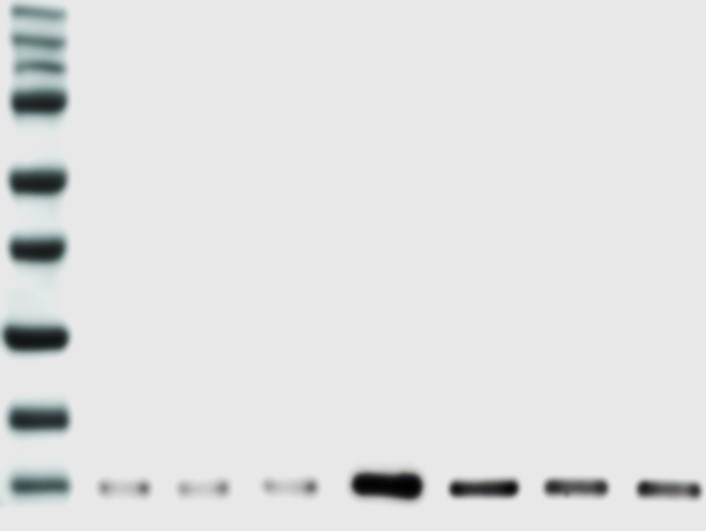

Supplement: Supplementary file 1 — (ZIP 222 KB) [file 12035_2025_5405_MOESM1_ESM.zip › western/CASPASE-1 1.jpg]

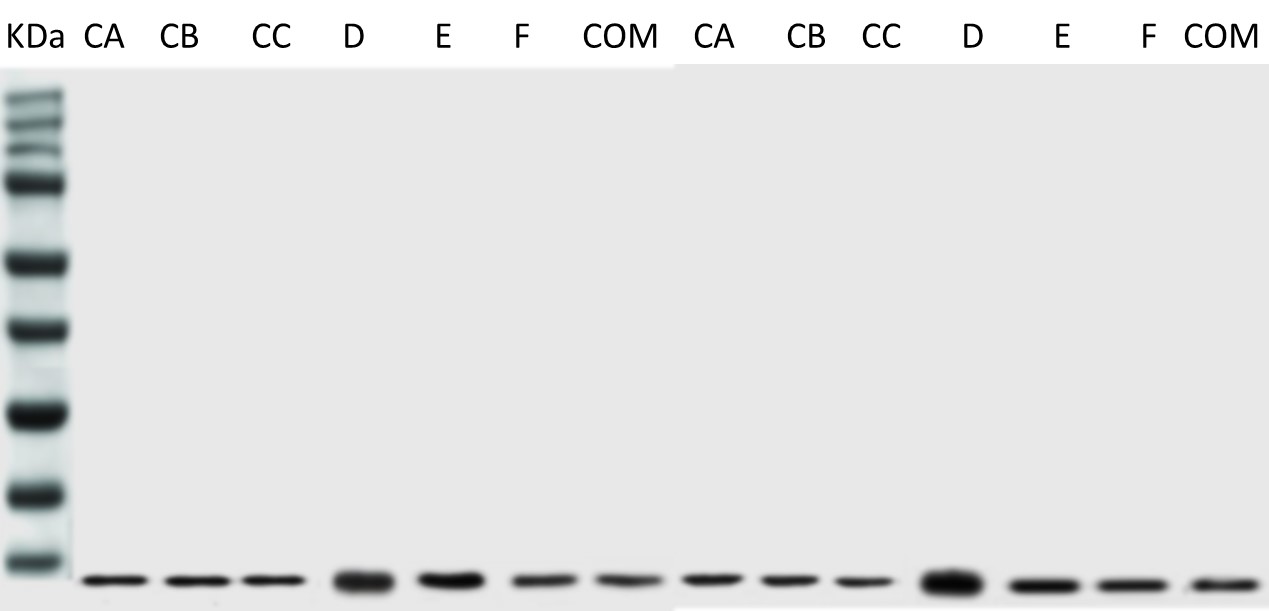

Supplement: Supplementary file 1 — (ZIP 222 KB) [file 12035_2025_5405_MOESM1_ESM.zip › western/CASPASE-1 2 AND 3.jpg]

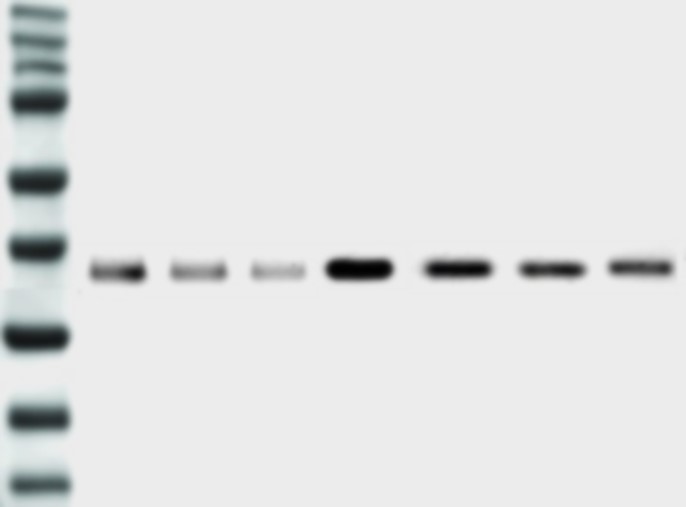

Supplement: Supplementary file 1 — (ZIP 222 KB) [file 12035_2025_5405_MOESM1_ESM.zip › western/GSDMD 1.jpg]

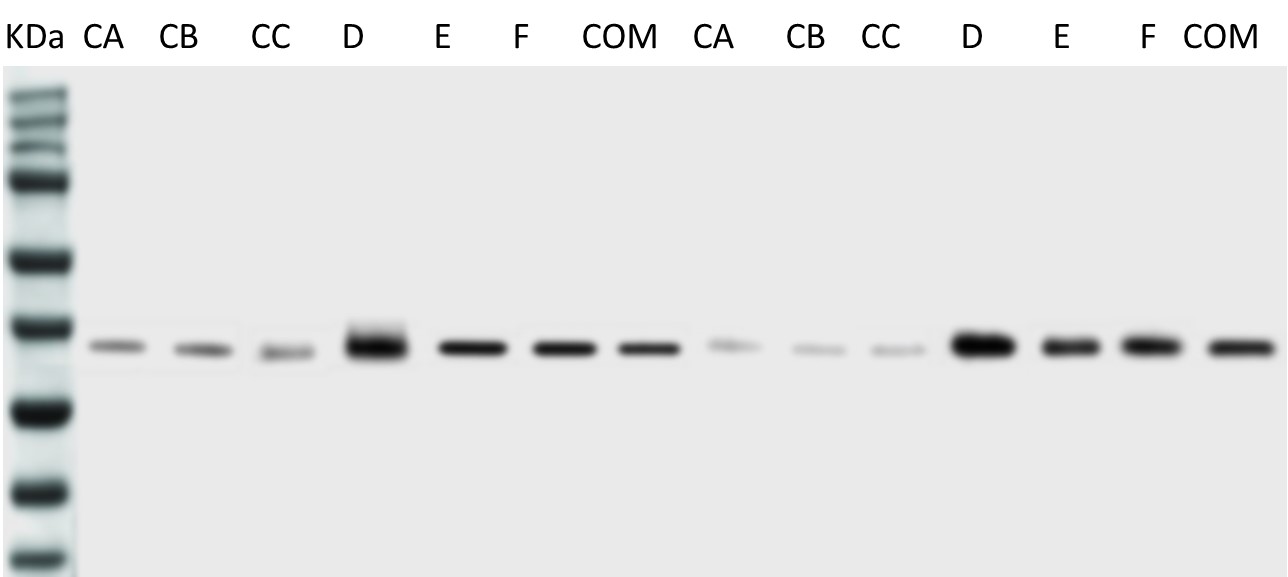

Supplement: Supplementary file 1 — (ZIP 222 KB) [file 12035_2025_5405_MOESM1_ESM.zip › western/GSDMD 2 AND 3.jpg]

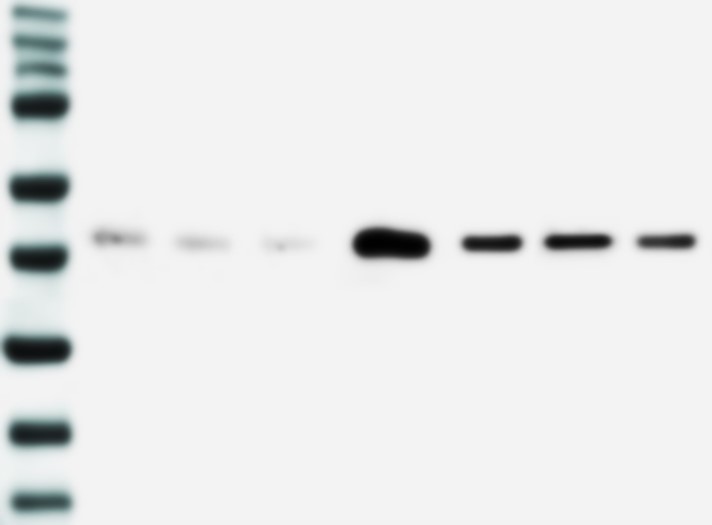

Supplement: Supplementary file 1 — (ZIP 222 KB) [file 12035_2025_5405_MOESM1_ESM.zip › western/NFKB 1.jpg]

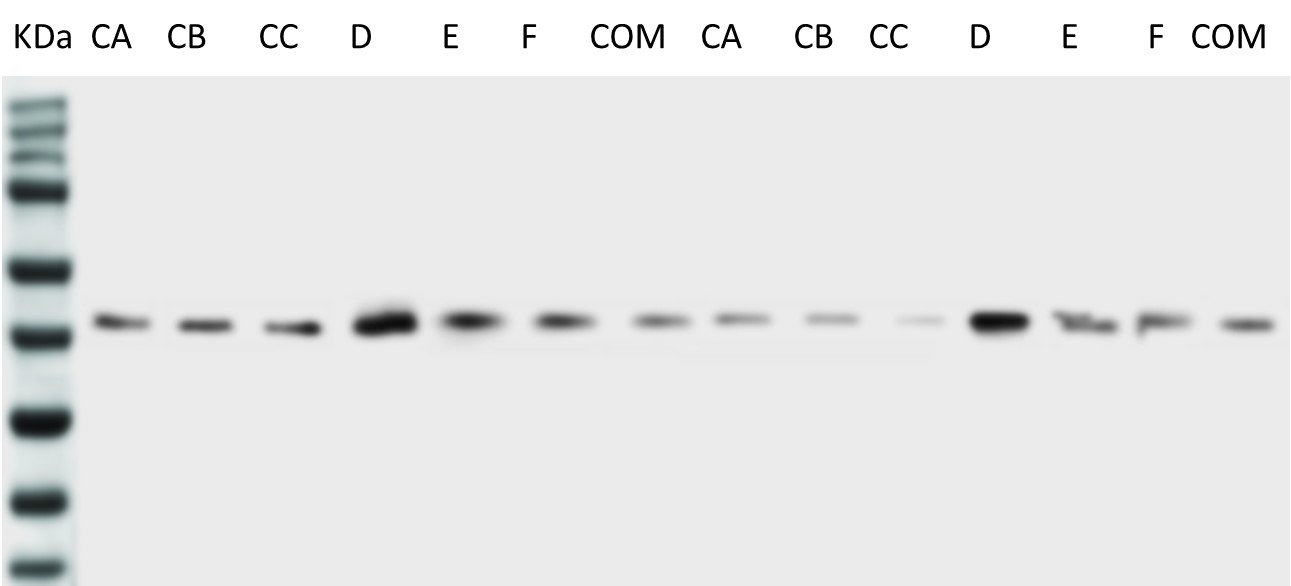

Supplement: Supplementary file 1 — (ZIP 222 KB) [file 12035_2025_5405_MOESM1_ESM.zip › western/NFKB 2 AND 3.jpg]

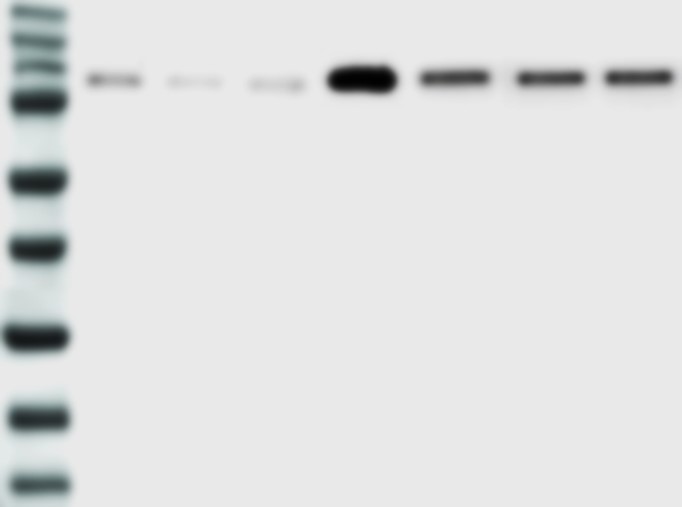

Supplement: Supplementary file 1 — (ZIP 222 KB) [file 12035_2025_5405_MOESM1_ESM.zip › western/NLRP3 1.jpg]

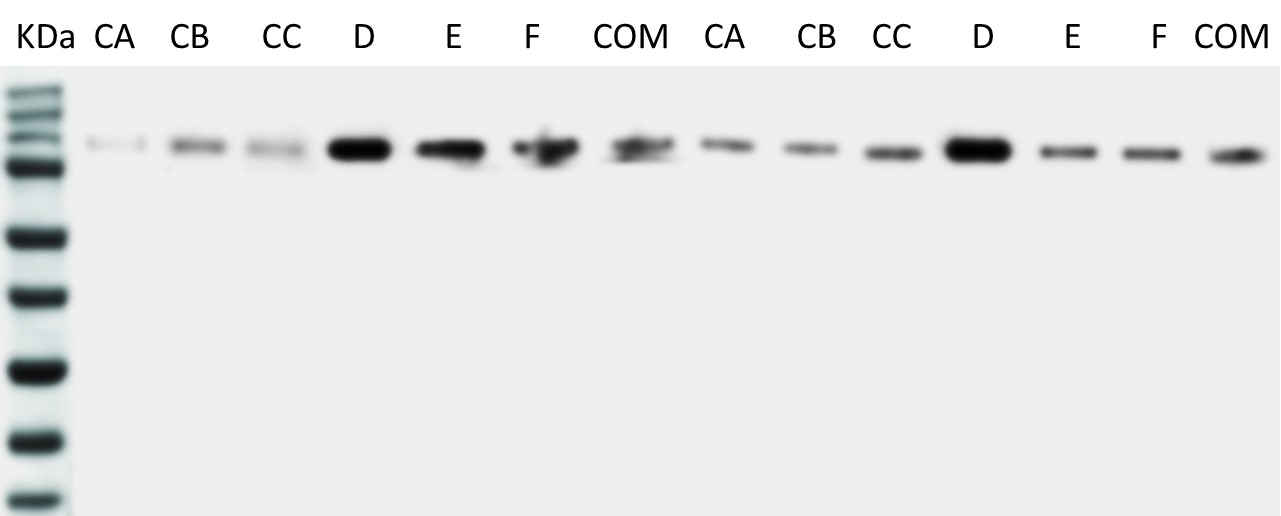

Supplement: Supplementary file 1 — (ZIP 222 KB) [file 12035_2025_5405_MOESM1_ESM.zip › western/NLRP3 2 and 3.jpg]

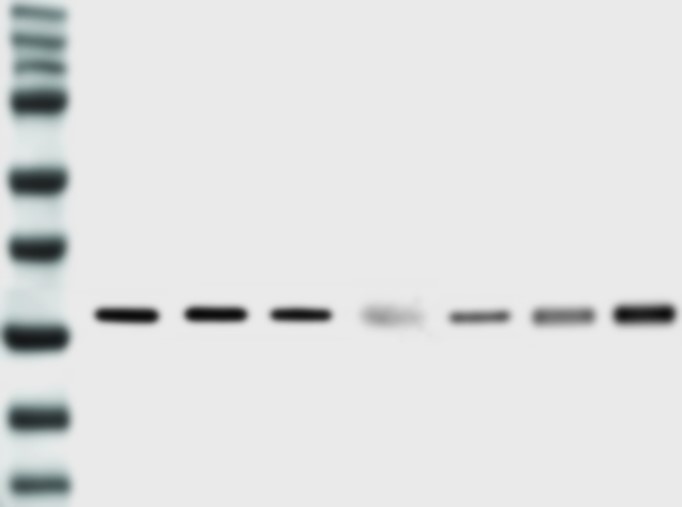

Supplement: Supplementary file 1 — (ZIP 222 KB) [file 12035_2025_5405_MOESM1_ESM.zip › western/TH 1.jpg]

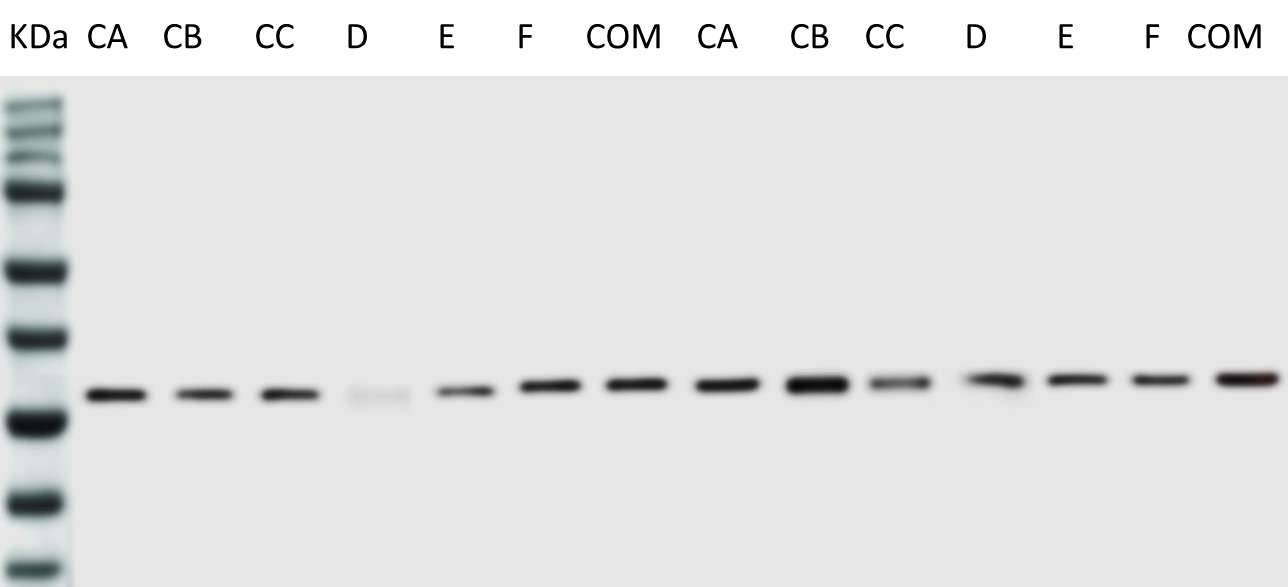

Supplement: Supplementary file 1 — (ZIP 222 KB) [file 12035_2025_5405_MOESM1_ESM.zip › western/TH 2 AND 3.jpg]
